# Essential Oil Fumigation Modulates Nutrient Content in Selected Mushrooms During Postharvest Storage

**DOI:** 10.3390/ijms26093939

**Published:** 2025-04-22

**Authors:** Małgorzata Grzyb, Kamil Szymczak, Radosław Bonikowski

**Affiliations:** Faculty of Biotechnology and Food Sciences, Institute of Natural Products and Cosmetics, Lodz University of Technology, Stefanowskiego 2/22, 90-537 Lodz, Poland; malgorzata.grzyb@p.lodz.pl (M.G.); kamil.szymczak@p.lodz.pl (K.S.)

**Keywords:** mushrooms, postharvest management, essential oils, fumigation, decay, food quality

## Abstract

Mushrooms are highly perishable, with a shelf life of up to three days. Considering their richness in nutrients and unique taste and aroma, extending their shelf-life presents a valuable field of exploration. Essential oil fumigation, already studied in plants, could effectively preserve mushroom quality by slowing the loss of nutrients. This study focused on the fumigation of two wild mushroom species, *Boletus edulis* and *Imleria badia*, as well as one cultivated species, namely, *Agaricus bisporus*, treated with *Foeniculum vulgare* (fennel) and *Picea abies* (spruce) essential oils. The fumigated mushrooms were stored for 4 days under non-optimal conditions and analysed for their content of free sugars and sugar alcohols, total FFA, composition of all fatty acids, vitamins, and ergosterol. The results were analysed using a linear model with three-way variable interactions, ANOVA type III, and multivariate PCA. The key findings indicated that spruce and fennel essential oil fumigation assured a high content of vitamin C (40 and 32.78 mg/100 g d.w.) and ergosterol (0.265 and 0.257 g/100 g d.w.) in *B. edulis* and a high content of vitamin D_2_ (1.94 and 1.55 µg/100 g d.w.) in *A. bisporus*. The results demonstrated that treating mushrooms with essential oils can effectively modulate the nutritional value loss.

## 1. Introduction

For ages, mushrooms have been an inseparable ingredient in European cuisine. In Poland, many traditional dishes include wild mushrooms, such as king bolete (*Boletus edulis*), bay bolete (*Imleria badia*), golden chantarelles (*Cantharellus cibarius*), or birch bolete (*Leccinum scabrum*). Their intense aroma, unique taste, and wide accessibility gave them a permanent place in traditional and modern cuisine.

Historically, mushrooms were not recognised as a nutritious food source or a significant reservoir of biologically active compounds with medicinal properties. However, recent studies highlight their beneficial composition. Mushrooms contain highly absorbable protein, making them vegan meat alternatives and a great source of unsaturated fatty acids [1]. While rich in polysaccharides, mostly β-glucans such as lentinan and schizophyllan, mushrooms possess immunological, anti-inflammatory, and hypoglycaemic activities [2]. They are also a good source of vitamins from group 2B, vitamins C and D_2_, and elements such as zinc, copper, iron, and manganese [1].

Therefore, given their significant nutritional and therapeutic potential, mushrooms can be used as functional foods or as a valuable source of nutraceuticals to support health and the quality of life. Their ability to produce a wide range of secondary metabolites with unique chemical structures and biological activities makes them a valuable source of chemical compounds.

Moreover, mushrooms are characterised by a high water content and a tendency to lose it just after harvesting. Typically, their shelf-life ranges from 1 to 3 days when stored at room temperature [3]. This short shelf-life is primarily due to the absence of a protective cuticle layer, making them vulnerable to physical damage, microbial contamination, and moisture loss. Eventually, it causes increased enzymatic activity, leading to accelerated lipid oxidation, an increased formation of free radicals, and a loss of nutrients and antioxidant compounds. Therefore, there is a growing need to develop preservation techniques that can extend the shelf-life of mushrooms, with essential oil fumigation emerging as one of the promising natural strategies.

Initially, fumigation was utilised for pest control purposes. It has evolved into a process where selected products are exposed to an atmosphere enriched with the vapours of various volatile substances, including essential oils. When applied to crops, this technique has revealed its capacity to inhibit microbial infections. It also has the potential to induce a substantial range of biochemical changes [4]. Applied in vapours, essential oils possess the capability to inhibit microbial growth [4] and increase enzymatic activity (such as superoxide dismutase or phenylalanine ammonia-lyase), which slows down the oxidative damage of health-promoting compounds [5].

The essential oils are natural mixtures of aromatic volatiles extracted from plants’ parts. The phytochemicals present in essential oils perform various functions in plants. For example, they play a crucial role in plants’ defence and signalling mechanism; they are characterised by various activities, such as antioxidant, anti-inflammatory, antibacterial, antiviral, and antifungal [6].

Among the vast diversity of essential oils, fennel and spruce oils are particularly noteworthy due to their distinct chemical compositions and biological activities, which have been the subject of increasing scientific interest. Fennel is a common name for a *Foeniculum vulgare* Mill plant species belonging to the *Apiaceae* (carrot) family. It is a widely cultivated, edible, and popular plant traditionally used in medicine. The scent of fennel essential oil results from the high content of *trans*-anethole (over 90%) in the composition and estragole; both compounds possess a sweet, phenolic, anise-like odour [7]. *Picea abies* (L.) H. Karst, known as Norway spruce, European spruce, and also Siberian pine, is a species of spruce that belongs to the Pinaceae family [8]. Its essential oil is most commonly derived from the needles of the tree. The main compounds present in the essential oil are α-pinene, β-pinene, camphene, and bornyl acetate [8].

Currently, only a few works focus on the fumigation of essential oils to prolong the shelf-life of mushrooms and delay the loss of some nutrients. All available studies focus on cultivated fungi species—*Agaricus bisporus* (button mushroom), such as [9,10], and *Lentinus edodes* (shiitake mushrooms), such as [11]. There is a lack of publications focusing on wild mushrooms such as *Boletus edulis* (king bolete) and *Imleria badia* (bay bolete). Those species are Poland’s two most popular fungi and are highly appreciated for their taste quality. Due to the uncontrolled growth conditions and diverse surface microbiota, wild mushrooms seem more complex. Thus, the effects of essential oil fumigation on such crops might be more challenging to predict. In the case of *Agaricus bisporus*, studies focus on essential oil fumigation, as well as other preservation methods utilising essential oils—active packaging [12], coatings [13], and nanoparticles [14]. These techniques have been shown to benefit the shelf-life of mushrooms significantly. However, their application is often limited by the need for advanced technologies, costly or difficult-to-obtain materials, and specialised equipment. As a result, their practicality for individual vendors and consumers remains restricted.

In contrast, essential oil fumigation is a simple technique that does not require specialised equipment and can be performed under almost any conditions. It makes it a more accessible and cost-effective alternative. Moreover, most research explores key enzyme activities, phenolic compound content, microbiological quality, and consumer acceptance. Their influence on the nutritional parameters of crops is still poorly explored.

Despite the promising potential of fumigation, only a single study has examined the effects of essential oil treatments on vitamins D_2_ and C [10]. There remains a significant gap in understanding how fumigation influences the overall nutrient composition of *A. bisporus*, such as vitamins and fatty acids. It highlights an important limitation in the current state of research, where the impact of essential oil fumigation on the actual nutritional quality of mushrooms remains insufficiently explored. Thus, there remains a significant gap in the literature regarding the impact of essential oil fumigation on the comprehensive nutrient profile of mushrooms, particularly when comparing wild versus cultivated species.

The main objectives of this study were to evaluate the impact of essential oil fumigation on the nutritional composition of three mushroom species (*Boletus edulis*, *Imleria badia*, and *Agaricus bisporus*) during postharvest storage. The species were chosen for their culinary utility as the most popular mushrooms in Polish cuisine. Specifically, the research aimed to (1) assess the effects of fennel and spruce essential oils on the retention of key nutrients, (2) determine whether the influence of fumigation varies between wild and cultivated mushrooms, and (3) analyse postharvest metabolic changes using multivariate statistical methods. The study aimed to provide insights into the potential application of essential oil fumigation as a natural preservation strategy for extending mushroom shelf-life while maintaining nutritional value. Our study addresses this gap by providing a detailed nutrient profiling of wild and cultivated mushrooms, analysing vitamins, fatty acids, and key bioactive compounds. This comprehensive approach enhances the understanding of fumigation effects and highlights the potential for developing accessible preservation strategies for a broader range of edible mushrooms.

## 2. Results and Discussion

The results presented in the following chapters mainly cover the data gathered before the essential oil treatment and after 96 h of storage; however, the analysis was performed after 48 h as well (except for free fatty acid and total fatty acid content). As those results lack statistical difference between them and the results at 0 or 96 h, they were omitted to assure the readability of the discussed results, although subsequent post hoc, ANOVA type III, and linear models incorporating three-way interactions were performed for all three time periods.

The concentration of essential oils used for the fumigation process was selected based on the preliminary experiment, where different concentrations (20–80 µL per box) were compared based on the overall appearance of the fruiting bodies. The choice of essential oil, concentration, and fumigation time was optimised during the preliminary experiments, and the parameters applied showed the best results. Also, the time of exposure was determined as the time of complete evaporation of essential oils from the filter paper, using the evaporation test procedure. The conditions of the storage after processing were chosen to fit the typical conditions in grocery stores.

### 2.1. Effects of Essential Oil Fumigation on Free Sugar and Free Sugar Alcohol Content

The free sugar and free sugar alcohol content is one of the most objective parameters of natural product deterioration. The polysaccharides in mushrooms undergo enzymatic breakdown due to the increased activity of their and spoilage microorganism enzymes, mainly cellulases, pectinases, lyases, polygalacturonases, and amylases [15]. The breakdown of the carbohydrate chains may result in the increased content of free sugars.

Some studies show that during postharvest storage, the content of soluble sugars in fruits increases—the vapours of methyl jasmonate caused a significant increase in sucrose, glucose, and fructose, however, a decrease in sorbitol concentration. It is associated with increased sucrose phosphate synthase gene expression, decreased acid invertase expression, and a lower sorbitol dehydrogenase gene expression [16].

The analysis showed a significant increase in all sugars and sugar alcohol content in all three species of fungi due to storage. For the glucose content, the most intensive rise was observable for *A. bisporus*, followed by the *B. edulis* fruiting bodies (Table 1). While D-glucose is a monosaccharide present in the β-glucans chains, it could be possible that those polysaccharides suffered intensely from the enzymatic hydrolysis during storage along with the glycogen, also present in mushroom cells. Both fumigated fruiting bodies of *I. badia* showed no significant differences compared to the time 0 samples. A different tendency was shown for the *A. bisporus* fruiting bodies, where the fennel-essential-oil-treated samples did not show a difference between those samples and the control ones. At the same time, spruce essential oil significantly inhibited the rise of free glucose content.

For *I. badia* mushrooms, the change in the glucose content was not observable for the vapour-treated samples, contrary to the non-treated ones. As the breakdown of polysaccharide chains is mainly the result of the enzymatic hydrolysis caused by the increased activity of spoilage microorganisms, such results may indicate selective microbial inhibition caused by the essential oils. For fructose content, which increased due to storage in all three species of mushrooms, all samples fumigated with essential oils showed different effects. Spruce essential oil caused a significant inhibition of the fructose content rise in *B. edulis* and *A. bisporus* and a decreased content in *I. badia.* On the other hand, fennel essential oil caused an even more significant increase in free fructose content in *B. edulis* while inhibiting it for the rest of the samples. While the inhibited decrease in the fructose content in fumigated mushrooms might be the cause of the suppression of the microbial growth causing the breakdown of sucrose or fructans, free fructose decrease might suggest activation of fructose-utilising pathways for metabolic energy in mushrooms. However, the breakdown of sucrose is unlikely due to increased content in all the samples after 96 h of storage. Similar observations can be made for xylose, whose accumulation may suggest the degradation of hemicellulose and its inhibition in spruce essential oil fumigation. It is most likely the result of monoterpene-vulnerable microorganisms.

Another sugar present in the studied mushrooms is trehalose, which is known as a stress-response disaccharide that can accumulate under abiotic stresses, such as desiccation. It results from the transcriptional activation of its biosynthesis pathways [17]. On the other hand, the increased trehalose content might be associated with the plant–pathogen interactions. It was shown that trehalose biosynthesis is associated with the initial plant infection. At the same time, its mobilisation is involved in virulence-associated functions that follow host colonisation in the case of *Magnaporthe grisea* infection of rice [6]. The significant increase in the trehalose content due to storage in both control and fennel-essential-oil-treated samples indicates the activation of stress-response mechanisms. In contrast, the decrease in the spruce-essential-oil-fumigated samples might result from the suppressed metabolism of mushrooms.

As presented in Figure S1 in Supplementary Materials, trehalose contributes to the largest share of all free sugars in all three fungi species. While the overall contribution in *Imleria badia* mushrooms is quite stable, for *Agaricus bisporus*, one can observe the increased share of glucose to the detriment of trehalose share during the storage. A similar observation can be made for spruce-essential-oil-fumigated king boletes in contrast to the other two variants of this mushroom, where the share of glucose decreased, changing the percentage distribution of free sugars towards trehalose.

Concerning free sugar alcohols, for the analysed fruiting bodies, the prevalent sugar alcohol is mannitol (Table 2), which, together with sorbitol and similarly to trehalose, is an indicator of metabolic stress and a key osmoprotectant [18]. The compounds present in essential oils, such as *trans*-anethole and *d*-limonene in fennel essential oils, and monoterpenes, such as bornyl acetate and α-pinene, although possessing antimicrobial properties, may also induce stress-related mechanisms. Such stress may cause an increased synthesis of sugars and sugar alcohols, which is observable for mannitol in *B. edulis* and *A. bisporus* fumigated with fennel essential oil and sorbitol in *B. edulis* treated the same way. Some compounds of essential oils, such as carvacrol, might cause an adaptive response to oxidative and osmotic stress. It activates excessive defence mechanisms such as accumulating osmolytes like sugar alcohols to stabilise the cellular structures [18,19,20].

The bar plot presented in Figure S2 in Supplementary Materials shows that sorbitol makes a noticeable contribution to total free sugar alcohols in *Boletus edulis* and *Agaricus bisporus* mushrooms; however, in *Imleria badia*, it was not detected. The contribution of free sugar alcohols in analysed mushrooms was relatively stable, with the highest deviations of sorbitol in king boletes and button mushrooms and arabitol and erythritol in bay boletes, where its contribution increased over storage in all the samples.

To better understand the influence of essential oil fumigation and storage time on the stability of polysaccharides in mushrooms, a linear model analysis supported by ANOVA III was applied (Supplementary Materials, Tables S1 and S2). The results show a high significance of storage time overall for free sugars and free sugar alcohols in fungi species, proving the adverse effects of postharvest storage on the quality of mushrooms regarding polysaccharide stability. Although the parameter Variant presents no statistical significance, one should note that it covers the measured sugars and sugar alcohol content after 96 h from fumigation treatment and the time 0—before the treatment. Hence, more appropriate parameters for interpretation are Variant:Time and VariantOil:Time96 interactions. The high statistical significance of Variant:Time—key interaction—indicates an effective influence of vapour treatment on the time impact on free sugar and free alcohol content. Thus, it may be concluded that essential oil fumigation has an undoubtful effect on modifying the undesirable changes in mushroom tissues. Concerning the influence of specific variants on the time impact on individual sugar and sugar alcohol content, it can be observed that neither fennel nor spruce essential oil significantly modifies the xylose content over storage time, and spruce essential oil has not been effective in inhibiting the increase in free sugars content apart from fructose and glucose. Based on Fungus and Fungus:Time effects, it is evident that different fungi species have different contents of specific sugars and sugar alcohols and suffer differently from postharvest storage.

### 2.2. Effects of Essential Oil Fumigation on Total and Free Fatty Acid Composition

Fatty acids are important in human organisms as they contribute to muscle contraction as structural cell membrane components and are responsible for organisms’ metabolism. Notwithstanding is the fact that the distribution of saturated, monounsaturated, ω-3 polyunsaturated, and ω-6 polyunsaturated fatty acids influences the exact properties of lipids [21]. Polyunsaturated fatty acids possess several health-promoting potentials, such as preventing cardiovascular diseases, breast and prostatic cancers, inflammations, neurological disorders, or visual system diseases [22]. Saturated fatty acids, on the other hand, are considered hazardous to human health and contributors to atherosclerotic cardiovascular diseases due to the increase in low-density lipoprotein cholesterol. Therefore, most health authority organisations recommend limiting the content of saturated fatty acids in the diet [23]. Studies, however, show that saturated fatty acids can play a crucial role in the biological functions of organisms. For example, myristic acid plays an important role in protein activation and potentially regulates the bioavailability of polyunsaturated fatty acids [24]. Some saturated fatty acids—mainly medium-chained—may influence metabolic processes, such as apoptosis and fat deposition [24].

Among all fatty acids, PUFAs are the most susceptible to degradation during storage. For example, the research done by Chaula et al. [25] showed that storage of sun-dried sardines for 21 days at ambient temperature caused an SFA content drop of 30% and a PUFA content drop of 65%. During the storage of crops, the activity of lipoxygenase, which is the enzyme responsible for catalysing the bioxygenation of polyunsaturated fatty acids, may be enhanced. This is primarily due to oxygen exposure, increased temperature, and water loss [26]. As a result, the balance of keeping the controlled level of lipoxygenase is disturbed, which may lead to an increased oxidation of PUFA, which eventually causes a decrease in its content [26]. The content of lipids may decrease during storage mainly due to the oxidation of the lipids, which leads to the accumulation of hydroperoxides. Eventually, it causes a decomposition of oxidation products, like alcohols, aldehydes and ketones, and free fatty acids [27]. The enhancement of the lipase activity during the storage catalyses the triglyceride hydrolysis, increasing the free fatty acid content. Such an effect is associated with increased crop water loss during postharvest storage [28]. Also, some preservation methods applied for mushrooms may result in accelerated fatty acid composition change.

Concerning the influence of postharvest treatments on mushrooms, conventional high-temperature drying and freezing can result in excessive degradation of essential fatty acids [29]. Some modern techniques, such as gamma and electron-beam irradiation, although showing a positive influence on some nutrients in mushrooms, decreased the unsaturated fatty acid contents [30]. As far as we know, there are no studies about the effects of essential oil fumigation or other essential-oil-utilising techniques on fatty acids in mushrooms.

Although mushrooms are low in calories due to a relatively small lipid fraction (Table 3, Table 4 and Table 5), they are rich in unsaturated fatty acids. Of the three species discussed in this research, the richest in fatty acid is *I. badia*, which has a total fatty acid content of about 1.380 g/100 g, while for *B. edulis*, it was about 1.122 g/100 g and for *A. bisporus* 1.025 g/100 g (Table 4). The most abundant fatty acid in all samples was linoleic acid (C18:2), constituting from 50% (*A. bisporus*—Table 5 and *B. edulis*—Table 3) to 60% (*I. badia*) of the total composition, followed by oleic acid (C18:1) constituting from about 17% (*I. badia*) to almost 30% (*B. edulis*). Both fatty acids constitute more than 80% of the total fatty acid composition in the discussed mushrooms. Saturated fatty acids comprise about 18% to 25% of all fatty acids, which results in a high UFA/SFA ratio equal to 4.7 in *Boletus edulis*, 3.3 in *Imleria badia* and 2.9 in *Agaricus bisporus*. It shows that wild mushrooms are a richer source of unsaturated fatty acids than cultivated ones. Although linoleic acid is the only ω-3 fatty acid present in the samples, its high concentration causes a high proportion of ω-3 to ω-6 fatty acid. From the saturated acids, the highest concentration is palmitic acid (over 10% in *B. edulis*, 16% in *I. badia* and 11% in *A. bisporus*), followed by stearic acid (about 3% in B. edulis, 5% in I. *badia*, and over 6% in *A. bisporus*). *Boletus edulis* fruiting bodies exhibited the lowest content of saturated fatty acids, accounting for approximately 17% of the total fatty acid composition, compared to 23% in *I. badia* and 25% in *A. bisporus*. In *B. edulis*, four monounsaturated fatty acids were detected: palmitoleic, heptadecenoic, oleic, and gondoic acids. In contrast, the other two species lacked heptadecenoic acid but contained erucic and nervonic acids, though these were present in very low concentrations (approximately 0.01–0.03% of total fatty acids). These results are partially consistent with the existing literature. Gałgowska et al. [21] and Heleno et al. [31] also reported that linoleic acid is the predominant fatty acid in *B. edulis* and *I. badia*, followed by oleic acid. However, Heleno et al. identified fewer fatty acids in their samples compared to this study, and the concentration of palmitic acid was lower than in the present analysis. Conversely, Gałgowska et al. reported similar values for these significant fatty acids, though their study’s overall distribution of PUFA, MUFA, and SFA shifted towards a higher MUFA content. As highlighted by Gałgowska et al., samples of the same species collected from different regions can exhibit considerable variation in fatty acid composition, which strongly shows the influence of environmental factors on lipid profiles.

All the samples suffered a loss in total fatty acids content—from 45% in fumigated samples to 56% in non-fumigated ones for *B. edulis*. Also, a loss from 28% in the fennel-essential-oil-fumigated sample and 34% in the spruce-essential-oil-fumigated sample to 46% in the control sample was observed for *I. badia*. For *A. bisporus*, it is shown that the level of total fatty acid decrease was equal to 28% in the fennel-essential-oil-fumigated sample and 35% in the spruce-essential-oil-fumigated sample, while it was about 48% in the control samples of *A. bisporus*. King boletes were then most susceptible to fatty acid content loss due to the postharvest storage. Essential oil fumigation significantly reduced its decrease, with fennel essential oil being most effective in all three fungi species. The most minor differences between the results after 96 h of storage are observable for saturated fatty acids, while they are the strongest for polyunsaturated fatty acids.

What is more, fumigation showed the weakest effects on this species as the fatty acids content decreased by relatively −45% and −46%, while for *I. badia*, it was −27% and −34% and for *A. bisporus* −28% and −35% for fennel essential oil and spruce essential oil, respectively. Postharvest storage caused the loss of long-chained unsaturated fatty acids—erucic and nervonic in *I. badia* and *A. bisporus*—with only fennel essential oil being able to cause partial preservation. Although essential oil fumigation reduced the changes occurring in the samples, the UFA/SFA ratio decreased, showing that saturated fatty acids are much less vulnerable during storage.

Nevertheless, this ratio remained higher for fumigated samples than for control ones except for *I. badia* fumigated with spruce essential oil, where the result is not significantly different than for the control sample. The UFA/SFA ratio does not show significant differences between the two essential oils in wild mushrooms, while for the *A. bisporus* samples, spruce essential oil showed the highest ratio. The results might suggest a better ROS scavenging potential of fumigated samples than control ones, probably resulting from mild metabolic stress induced by exposure to volatile compounds. It might have triggered the activation of the stress-response pathways to produce antioxidants, including enzymatic (e.g., superoxide dismutase and catalase) and non-enzymatic components (e.g., phenolic compounds and ascorbic acid). Eventually, it may cause the stabilisation of cell membranes and the minimisation of lipid peroxidation, thereby preserving the integrity of unsaturated fatty acids. Furthermore, some volatile compounds in essential oils may denaturate and inhibit the activity of lipoxygenase—the enzyme causing the degradation of fatty acids [32].

**Figure 1:**
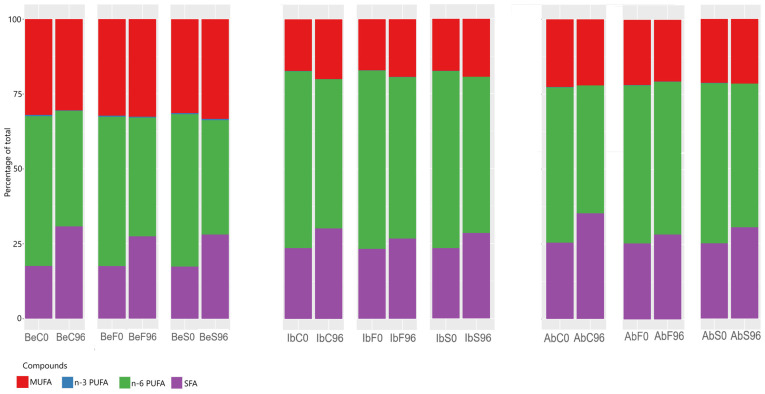
Bar plot presenting the Z-scored distribution of fatty acid groups within samples. Be, Ib, and Ab are *B. edulis*, *I. badia* and *A. bisporus*. C, F, and S represent control samples, fennel-essential-oil-fumigated samples, and spruce-essential-oil-fumigated samples.

Not only did the absolute contents of specific fatty acids change over storage but also the general profile of fatty acids and distribution of specific groups. Figure 1 shows that the monosaturated fatty acids’ share in the total fatty acid composition is stable, presenting only mild variations—the strongest for king boletes. On the other hand, the distribution between n-6 PUFA and SFA shifted strongly towards saturated fatty acids, which complies with the general conclusion that saturated fatty acids are much more stable during storage than polyunsaturated ones. Essential oil treatment has alleviated those changes in all three fungi species, with fennel essential oil being slightly more effective.

Similar to the composition of total fatty acids, linoleic and oleic acids contribute mainly to free fatty acid compositions. The sum of total free fatty acids brings about 10–15% of all fatty acids before storage. After 96 h, the content of free fatty acids increased rapidly by 69% in *B. edulis* (Table 6), 114% in *I. badia* (Table 7), and 84% in *A. bisporus* (Table 8). The highest relative increase was observed in myristic and palmitoleic acid (*B. edulis*, where the concentration of myristic acid increased from 0.53 mg/100 g d.w. to 2.11 mg/100 g d.w. and the concentration of palmitoleic acid increased from 0.69 mg/100 g d.w. to 2.94 mg/100 g d.w.), pentadecanoic acid (*I. badia*, where the concentration of this fatty acid increased from 0.68 mg/100 g d.w. to 2.93 mg/100 g d.w.), and gondoic acid (*A. bisporus*, where the concentration of this fatty acid increased from 2.49 mg/100 g d.w. to 10.56 mg/100 g d.w). Due to the postharvest storage, the share of free fatty acids in all fatty acid contents decreased intensely to 45–61%, depending on the species. It shows that the soft structure of mushrooms causes a quick deterioration and intense loss of nutrients. Essential oil fumigation inhibited this rise in all three fungi species. The most significant influence of volatiles was observed in bay boletes, where the increase in free fatty acid content of fennel-essential-oil-fumigated mushrooms was equal to 41%. This essential oil seemed most effective in bay boletes and button mushrooms; however, it showed smaller efficiency in king boletes than spruce essential oil. Essential oil treatment inhibited the breakdown of the lipids containing palimitelaidic and margaric fatty acids in *B. edulis*, and those were not detected in those samples after 4 days of storage, contrary to other mushroom species. The inhibited activity of endogenous lipases in mushroom tissues might influence the decreased free fatty acid content rise.

According to the linear model and ANOVA III, time has a high impact on increasing all the free fatty acids, indicating that postharvest storage truly influences the stability of lipids in mushrooms (Supplementary Materials, Table S3). Moreover, the fumigation with essential oils effectively modifies the changes occurring in analysed species, apart from the free behenic acid and free myristic acid, which rise during storage and have not been impacted in any way by the fumigation. It can be concluded that the content of free saturated acids is much harder to manipulate by postharvest treatments. It is worth noting that the content of free margaric acid, absent in fresh mushrooms before storage, does not depend on the mushroom species, and the content of free palmitic acid changes over time in a fungus-independent manner.

Regarding total fatty acids present in the samples, one can observe a lack of significance of Variant:Time interaction on 4 out of 9 saturated fatty acids (Supplementary Materials, Table S4), supporting the hypothesis of the limited impact of essential oil on modifying the specific saturated fatty acids content. However, this interaction shows significance on total SFA. Overall, the fumigation itself, as well as specifically fennel essential oil and spruce essential oil, had a significant impact on the change of sums of SFA, MUFA, and PUFA groups, as well as the total fatty acid content and the UFA/SFA ratio that happened due to storage.

### 2.3. Effects of Essential Oil Fumigation on Tocopherol, Ergosterol, and Vitamin D_2_ Content

Tocopherols are a group of compounds included in the vitamin E profile. Those are the secondary metabolites of organisms that protect cellular membranes from the oxidative effect of reactive oxygen species occurring in plants and mushrooms due to metabolic degradation over storage. As fat-soluble primary antioxidants, they play a key role in intercepting the lipid hydroperoxide over the decomposition processes. Thus, they protect especially the unsaturated lipids from oxidation and prevent increased content of free fatty acids. The radical scavenging ability of tocopherols results from the fused chroman ring system and phytyl side chain, ensuring the optimal position of the reactive centre location [33]. Besides their protective role to plants, tocopherols exhibit health-promoting properties, such as anti-inflammatory and anti-nitrative, and are associated with preventing cardiovascular, neurodegenerative, and cancer diseases [34].

The concentration of tocopherols in food products is directly connected to their quality as the deterioration causing lipid oxidation results in unpleasant odour and taste development. Such changes disqualify the product not only because of its diminished nutritional values but also because of its poor organoleptic properties [35].

Since tocopherols are highly perishable and vulnerable to oxidation due to reactive oxygen species, introducing additional antioxidative compounds might preserve the levels in plants and mushrooms. Fennel and spruce essential oils possess high antioxidant activity due to the presence of phenylpropanoid derivatives (trans-anethole, estragole [7], and fenchone [36] for fennel essential oil) and terpenoid derivatives (α- and β-pinene [37] and bornyl acetate [38] for spruce essential oil). Hence, their vapours might effectively mitigate oxidative stress, leading to tocopherol degradation.

All forms of tocopherols suffered a significant loss during the postharvest storage of all three species of fungi. It can be observed that α-tocopherol, which is the most active form, and β-tocopherol dropped the strongest for all the control samples of mushrooms. Fennel essential oil vapours effectively retained high concentrations of γ-tocopherol, while spruce essential oil effectively reduced the drop of α-tocopherol in *Boletus edulis* mushrooms (Table 9). Although spruce essential oil did not significantly inhibit the drop of β- and γ-tocopherol content, the sum of all tocopherols was much higher than in the case of control samples. Both essential oils protected about 15% of tocopherols in *Boletus edulis* mushrooms. On the contrary, spruce essential oil vapours caused the excessive loss of tocopherols in *Imleria badia* mushrooms, especially regarding α- and δ-tocopherols. As *Imleria badia* mushrooms are the richest in tocopherols among those three species, one might suggest that their antioxidant strategies rely on tocopherols stronger than in the case of other species. Hence, the stress-induced response to the spruce essential oil compounds might result in accelerated tocopherols loss. It might also disrupt and decrease the activity of enzymes responsible for tocopherol regeneration (like tocopherol cyclase) or decrease the content of tocopherol-regenerating antioxidants, such as ascorbic acid or coenzyme Q [39]. It can be suspected that similar observations will be made for other fat-soluble nutrients, such as ergosterol or ergocalciferol.

Ergosterol (ergosta-5,7,22-trien-3β-ol) is a sterol responsible for the fungi cell membrane integrity of antioxidant properties, such as inhibiting lipid peroxidation or scavenging the intracellular radical oxygen species [40]. Moreover, ergosterol takes part in the signalling pathways of suppressing the inflammatory mediator’s expression and exhibits anti-inflammatory properties [40]. Finally, it was shown for its neuroprotective and anti-hepatic steatosis effects and anti-diabetic and anticancer activity [40]. It is crucial for fungi physiology because ergosterol is the precursor in the vitamin D_2_ synthesis pathway through conversion under ultraviolet radiation [41]. Ergocalciferol, or vitamin D_2_, is one of two primary forms of vitamin D. Although having a shorter action duration and less potent than vitamin D_3_, it is the only non-animal-derived form of this vitamin, making it the perfect substitute for vegans. It has a pivotal role in enhancing the absorption of calcium and phosphorus and regulates bone mineralisation [42]. Also, it reduces the risk of osteoporosis by maintaining skeletal health and modulates the immune system by regulating inflammatory responses of the organisms [43]

**Figure 2:**
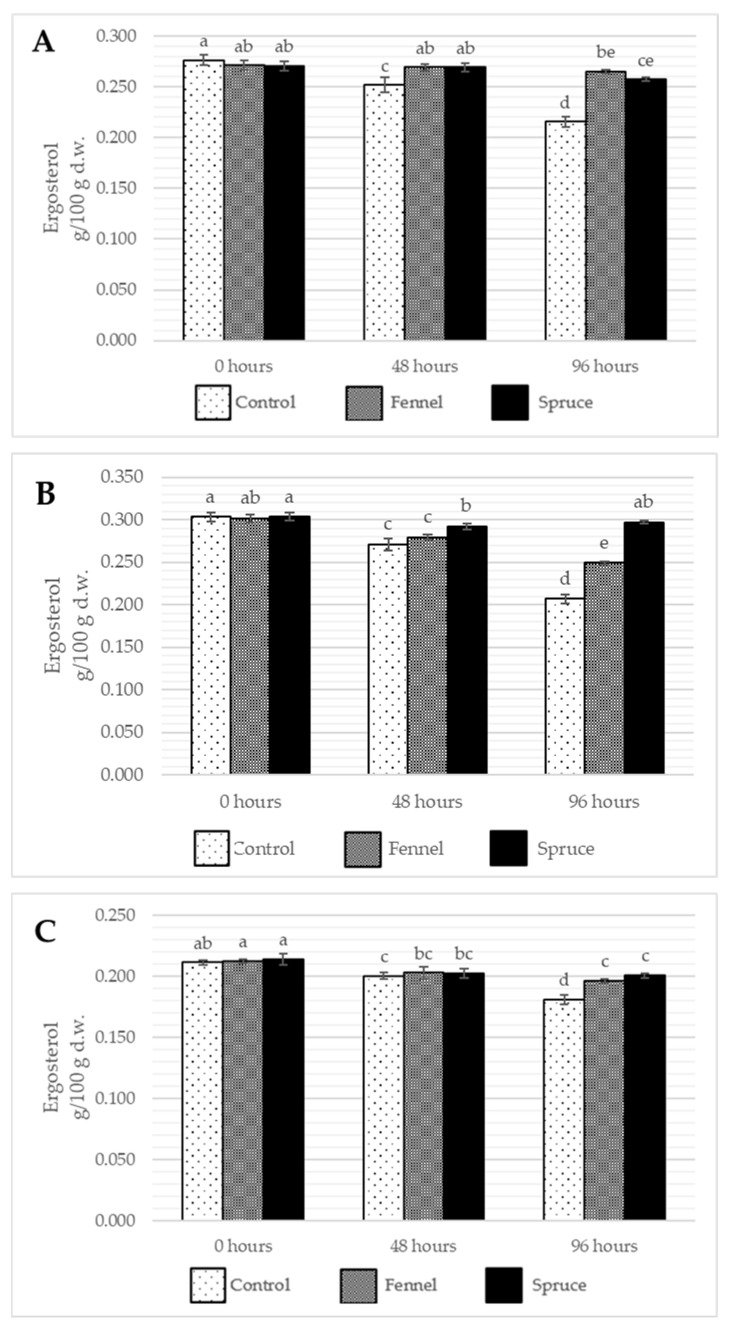
The average content of ergosterol (g/100 g d.w) in *B. edulis* (**A**), *I. badia* (**B**) and *A. bisporus* (**C**) before the treatment and storage (0 h) and after 48 and 96 h of storage. Control, Fennel, and Spruce stand for samples without treatment, fumigated with fennel essential oil and fumigated with spruce essential oil, respectively. Means are averaged values of three replicates ± standard deviation (vertical bars). Values for all time points among one species with the different letters are significantly different (*p* ≤ 0.05).

**Figure 3:**
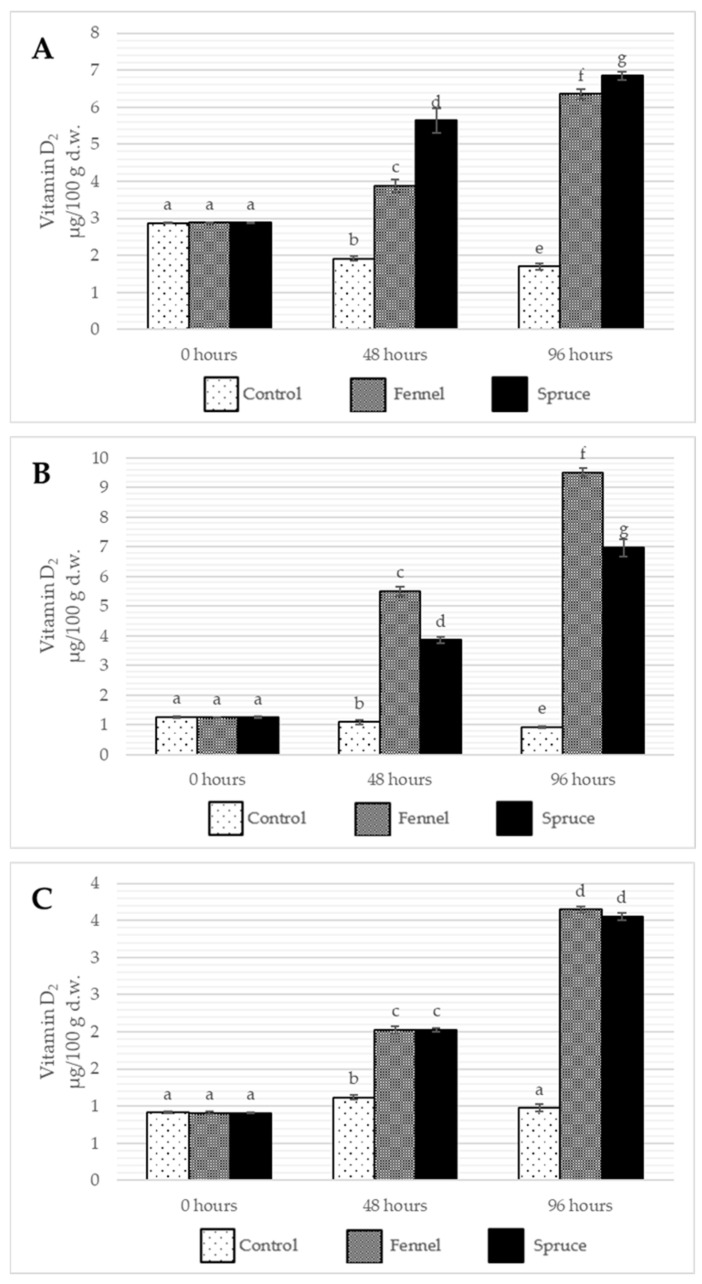
The average content of vitamin D_2_ (µg/100 g d.w) in *B. edulis* (**A**), *I. badia* (**B**) and *A. bisporus* (**C**) before the treatment and storage (0 h) and after 48 and 96 h of storage. Control, Fennel, and Spruce stand for samples without treatment, fumigated with fennel essential oil and fumigated with spruce essential oil, respectively. Means are averaged values of three replicates ± standard deviation (vertical bars). Values for all time points among one species with the different letters are significantly different (*p* ≤ 0.05).

The results showed that the content of both ergosterol and ergocalciferol decreases over time, probably linked strictly to its oxidation due to microbial infection (Figure 2 and Figure 3). The fumigation of the samples preserved ergosterol content at a high level and slightly increased the concentration of vitamin D_2_ during storage, which might be caused by oxidative stress accelerating ergosterol conversion. Among all the discussed species, *I. badia* suffered the highest drop of ergosterol content in control samples, with fumigation with spruce essential oil showing the most intense inhibition of the ergosterol loss (no significant differences between the ergosterol content before storage after 96 h from the treatment). The other two species had no significant differences between the essential oils used for the treatment. Also, due to postharvest storage, *A. bisporus* was found to be the least susceptible to losing ergosterol. After 48 h of storage, there were little to no differences in the ergosterol content. When it comes to ergocalciferol, its content increased due to fumigation in all the samples, while the highest was observed for *I. badia* and *A. bisporus* fumigated with fennel essential oil (rise from 1.25 to 9.51 µg/100 g and from 0.91 to 3.65 µg/100 g for the respective species). For *B. edulis*, spruce essential oil showed a more substantial effect, while for *I. badia*, no significant differences between the treatments were observable after 48 and 96 h from the treatment. After 48 h from the treatment, the tendency to lose vitamin D_2_ in control samples and increase its content in fumigated samples was already observable. Due to their lipophilicity, essential oils may disrupt the membrane of mushrooms by increasing their permeability and access to UVB radiation [44]. Such observation is in line with Aly et al. [10] findings as the results of lemongrass and geranium essential oil fumigation caused an increase in ergocalciferol content in the button mushrooms immediately after the treatment. Although the vitamin D_2_ eventually decreased, the storage time was much longer (12 days) and stored at 4 °C, possibly in the dark, without access to ultraviolet radiation triggering the ergosterol conversion.

The enhanced stability of fat-soluble compounds—tocopherol, ergosterol, and ergocalciferol—observed in the fumigated mushrooms likely results from both antioxidant protection and membrane stabilisation effects induced by essential oils. Tocopherols are particularly vulnerable to lipid peroxidation in the presence of radical oxygen species. Components of essential oils can directly neutralise ROS or chelate transition metal ions, resulting in the mitigation of the oxidative stress in mushroom tissues [45]. Ergosterol and ergocalciferol are similarly susceptible to oxidation under postharvest conditions. Essential oil vapours may create a transient protective microenvironment, limiting oxygen diffusion and membrane permeability, thus reducing sterol degradation [46].

According to the linear model and ANOVA III test, the results indicate more substantial statistical evidence for the impact of time on other fat-soluble compounds in mushrooms compared to δ-tocopherol (Supplementary Materials, Table S5). Although the Variant:Time interaction shows statistical significance only in β-tocopherol, which indicates that fumigation does not modify the influence of time on the other tocopherol content, the sum of tocopherols is undoubtedly impacted. It may suggest that fennel essential oils influence β-tocopherol, which may mitigate its insignificant impact on other forms and the lack of statistical significance of spruce essential oil impact after 96 h from the treatment. These results and the Tukey-test post hoc analysis prove the prevalence of fennel essential oil in inhibiting the loss of tocopherol content in mushrooms over spruce essential oil.

When it comes to the influence of different parameters and their interactions on ergosterol and ergocalciferol content change, all show statistical significance, which strongly supports the hypothesis that essential oil fumigation modifies the changes occurring in mushrooms during postharvest storage. Like in the interpretation of the linear model and ANOVA III results of other result subchapters, different fungi species show significant differences in fat-soluble chemical content. Such content is modified in various ways over time in different fungi species.

### 2.4. Effects of Essential Oil Fumigation on Water-Soluble Vitamins

Water-soluble vitamins play an invaluable role in human organisms by maintaining the normal metabolic, energy, differentiation, and growth status of the cells [47]. Most water-soluble organisms must be introduced to humans from exogenous sources due to the human’s lack of synthesis ability [47]. In mushrooms, the most abundant vitamins are C and variants of vitamin B.

Vitamin C quickly degrades during storage as it is highly susceptible to temperature, oxygen exposure, and light. The degradation of ascorbic acid leads to the formation of dehydroascorbic acid and further breakdown products, such as 2,3-diketogulonic acid, which do not possess vitamin activity [48]. Even frozen mushrooms suffer significant vitamin loss—higher for the longer storage time [49]. There are some indications in the literature that essential oil fumigation might be effective in inhibiting the ascorbic acid loss over the storage period in strawberries [50], peaches, and nectarines [51] but also button mushrooms [10]. On the other hand, some articles show the increased loss of ascorbic acid due to essential oil vapours [52], which suggests that the effects of such treatment are hard to believe, especially concerning difficulties in selecting an appropriate essential oil and its concentration to avoid excessive stress induced in crops.

**Figure 4:**
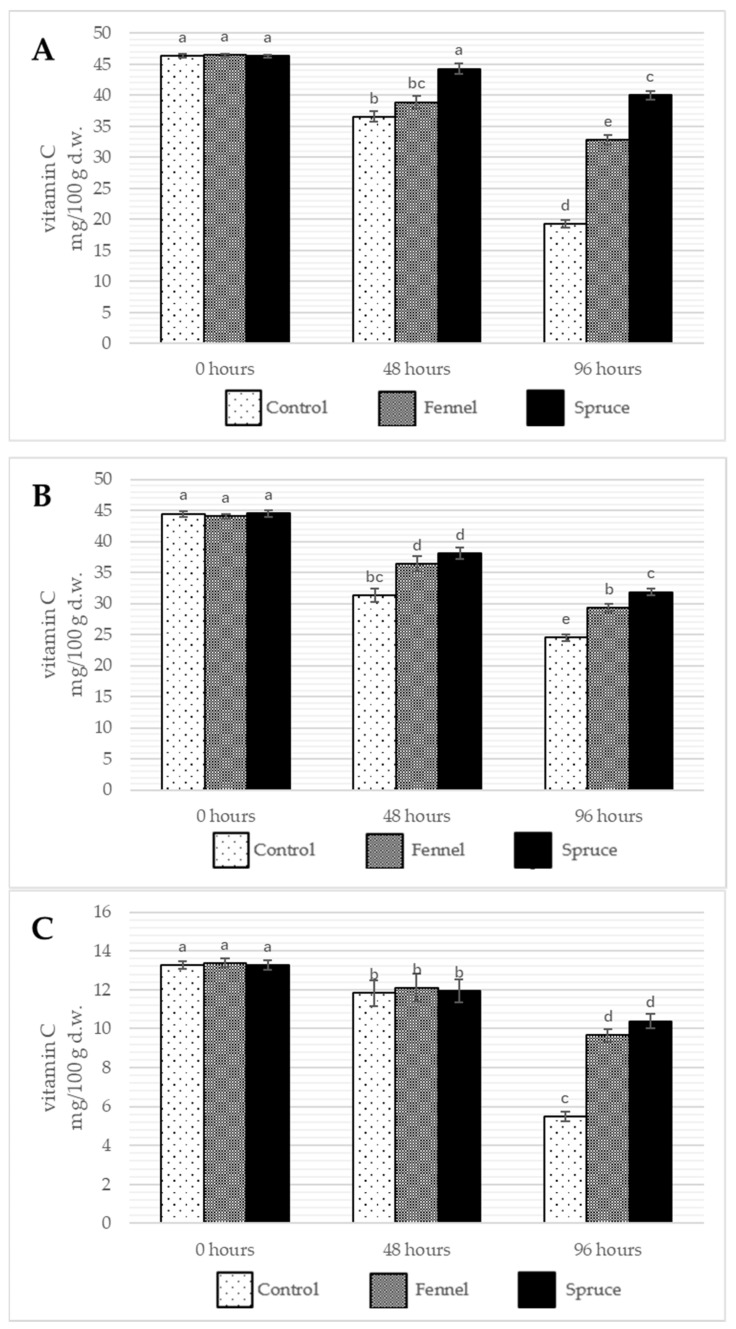
The average content of vitamin C (mg/100 g d.w) in *B. edulis* (**A**), *I. badia* (**B**), and *A. bisporus* (**C**) before the treatment and storage (0 h) and after 48 and 96 h of storage. Control, Fennel, and Spruce stand for samples without treatment, fumigated with fennel essential oil and fumigated with spruce essential oil, respectively. Means are averaged values of three replicates ± standard deviation (vertical bars). Values for all time points among one species with the different letters are significantly different (*p* ≤ 0.05).

The results of this experiment show that both essential oils effectively decreased the decease rate in vitamin C (Figure 4) and vitamin B_1_ content over the storage while having no significant effect on modifying the vitamin B_2_ loss (Table 10). The most substantial drop of the vitamin C content is observed for *B. edulis* mushrooms (from 44.47 to 24.05 mg/100 g d.w.) and for *A*. *bisporus* (from 13.27 to 5.50 mg/100 g d.w.). In all the cases, spruce essential oil showed more substantial effects than fennel essential oil, with *B. edulis* presenting the most intensive effect. After 48 h of the treatment, the drop in vitamin C content in *A. bisporus* mushrooms was similar in all the samples, and the results showed no significant differences between them. In the other two species, the tendency for the higher drop of vitamin C content in control samples was already observable and remained the same after the next 48 h. Moreover, the inconsistent effect of essential oil fumigation is observed for vitamin B_6_ content as in king boletes and button mushrooms, only fennel essential oil caused the reduced loss rate; in bay boletes, both essential oils accelerated the B_6_ vitamin decrease. Since vitamin B_6_ is susceptible to acidic pH, one may speculate that essential oils modified the surface pH of *Boletus edulis*, fruiting bodies, resulting in an increased loss of this vitamin. B vitamins and ascorbic acid are water-soluble, so one may expect its content to be much better preserved if the water loss in mushrooms is limited. Essential oils as hydrophobic mixtures may act as coating, creating a physical barrier that reduces crops’ water loss and modifies transpiration metabolism [52]. The results for vitamin C content are consistent with those published by Aly et al. [9], where geranium and lemongrass essential oil fumigation efficiently diminished the loss of ascorbic acid in *Agaricus bisporus* mushrooms. As for B vitamins, no other publications allow the comparison of the results. The observed preservation of vitamin C and B-group vitamin content in fumigated mushrooms may be attributed to the antioxidant properties of essential oil components such as *trans*-anethole and α-pinene. These compounds are known to inhibit oxidative stress by scavenging free radicals [53] and possibly suppressing the activity of oxidative enzymes like polyphenol oxidase and peroxidase. Additionally, essential oils may reduce microbial spoilage, indirectly slowing nutrient degradation. The possible mechanisms of the essential oils acting as preservatives are yet unknown and need to be further explored. There is a possibility that essential oils change the structure of mitochondria and modify enzymes and energetic substances in the respiratory chain; however, such mechanisms need further exploration [46].

The results of the linear model and ANOVA III tests (Supplementary Materials, Table S6) show a lack of an impact of fumigation on the vitamin B_2_ changes over time, unlike for other water-soluble vitamins. Hence, unlike other vitamins, the riboflavin loss due to postharvest storage cannot be inhibited by those two essential oils in those experimental conditions. Interestingly, although the content of this vitamin is statistically different over different fungi species, unlike in other analyses, the Fungus:Time interaction result shows that riboflavin content changes similarly with no statistically significant differences between the three species. Other water-soluble vitamins show statistical significance of parameters Time and Fungus as well as Variant:Time and VariantFennel:Time96, and VariantSpruce:Time96, suggesting the usefulness of essential oil vapours in modifying the nutrient loss.

### 2.5. Principal Component Analysis (PCA) of Samples’ Metabolic Profiles

PCA was performed to perform the exploratory data analysis. Components explains variance as follows: PC1—41.06%, PC2—27.71%, and PC3—13.78% of variance. Cumulatively, components 1–5 explain 93.92% of variance.

#### 2.5.1. Two-Dimensional Feature Analysis

**Figure 5:**
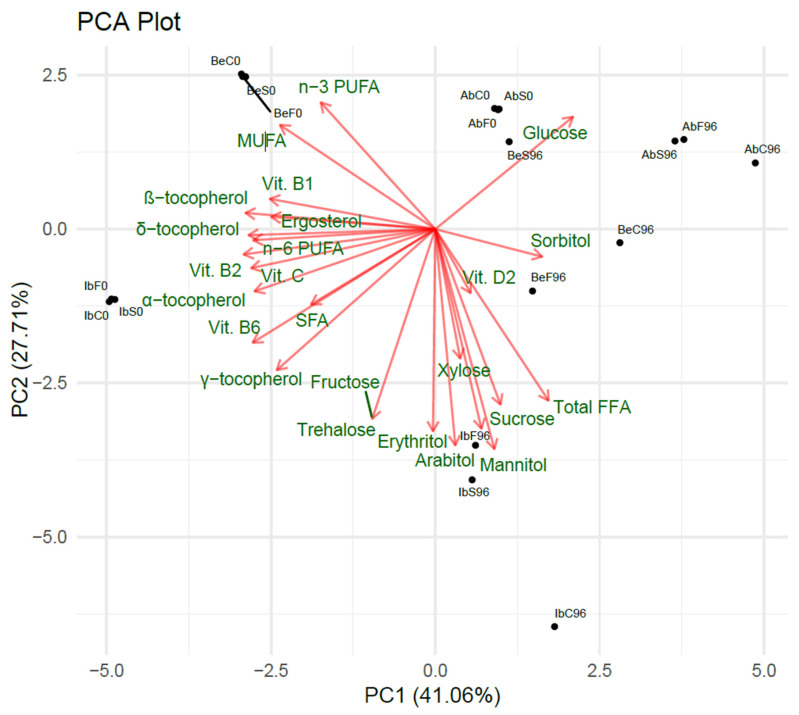
Two-dimensional plot of the PCA results obtained from metabolite data.

The PCA plot (Figure 5), together with the rotation values for features, indicate the following:

- Vitamin B_2_, vitamin B_6_, β- and δ-tocopherol, and vitamin C had the most substantial impact on PC1;
- Mannitol, arabitol, trehalose, erythritol, fructose, sucrose, and total FFA had the most substantial impact on PC2.

Moreover, the total FFA content correlates negatively with the total n-3 PUFA and total MUFA content in mushrooms; it correlates negatively with the total SFA and n-6 PUFA within the PC1, which explains the highest percentage of variance. Free glucose correlates negatively with SFA, vitamin B_6_, and γ-tocopherol, and free sorbitol correlates negatively with the vitamin B_1_, β- and δ-tocopherol, and ergosterol content. It supports the conclusion that storing mushrooms is associated with the loss of nutritional compounds, but it also leads to the breakdown of polysaccharides and triglycerides, which increase total free sugar, free sugar alcohol, and free fatty acid content. Interestingly, most free sugars and free alcohols significantly negatively influence principal component 2, except for sorbitol and glucose (and slightly fructose). In contrast, most vitamins negatively impact principal component 1, except for D2. Two significant feature clusters can be differentiated within the PCA loadings plot. In opposition to those clusters, two primary compounds are separated—glucose and sorbitol.

#### 2.5.2. Three-Dimensional Sample Distribution Analysis

**Figure 6:**
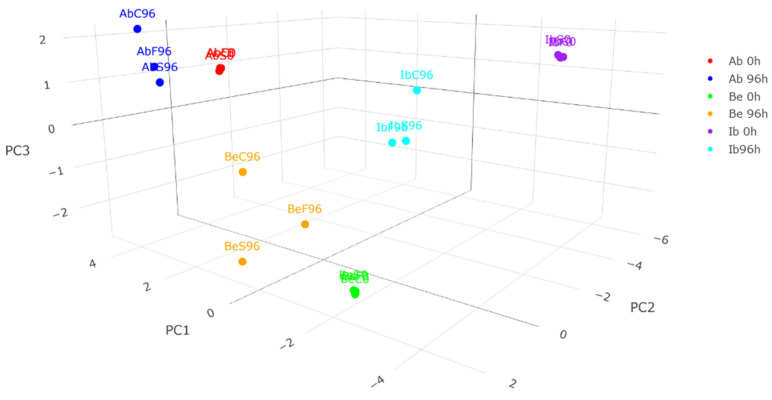
A projection of a three-dimensional plot of the PCA results obtained from metabolite data (a 3D file of PCA is available in Supplementary Materials as File S1).

The 3D PCA plot (Figure 6) shows the distribution of samples within three principal components. Principal component 1 differentiates samples before the postharvest storage from samples after the storage and different fungi species before the storage. As it is strongly negatively impacted by most of the vitamins, and samples after the storage are shifted towards positive PC1, it proves that the loss of those vitamins correlates with the long postharvest storage of mushrooms. Interestingly, it moderately differentiates control samples after 96 h of storage from fumigated samples, with control samples shifted stronger towards positive PC1, showing a positive influence of fumigation on keeping higher contents of tocopherols, vitamins from group B, vitamin C, ergosterol, and n-6 polyunsaturated fatty acids.

Principal component 2 does not visibly affect the distribution of *A. bisporus* samples within the storage time, while for wild mushrooms, the differentiation is much stronger—especially in the case of *I. badia* mushrooms. Since it is highly impacted by most of the free sugars, free sugar alcohols, and total FFA, it can be concluded that those metabolites did not affect button mushrooms highly, unlike wild mushrooms—both wild fungi species suffer intense changes of those metabolites, proving their vulnerability to changes of free compound content. Moreover, PC2 differentiates *I. badia* control samples after 96 h from the fumigated ones while differentiating spruce-essential-oil-fumigated *Boletus edulis* samples from the other two. Since free sugars, free alcohols, and free fatty acids impact PC2 negatively, and the spruce-essential-oil-fumigated sample is shifted towards the positive axis, it again proves that such treatment effectively inhibits the undesirable changes in mushrooms, as such observation can be made for all three species.

Principal component 3 has not been presented on three-dimensional sample distribution analysis since it explains 13.78% of the variance; it is worth mentioning in the case of sample distribution analysis since it gives additional information about the vitamin D2, n-3 PUFA, and xylose—so, the minor compounds in mushrooms—impact on the samples and SFA content. Those minor compounds impact PC3 negatively in opposition to SFA. This suggests that samples with a high SFA content would also have a low content of the other metabolites within principal component 3. PC3 differentiates control samples after the treatment from the fumigated samples—the latter being shifted towards negative values.

#### 2.5.3. Hierarchical Clustering Analysis

**Figure 7:**
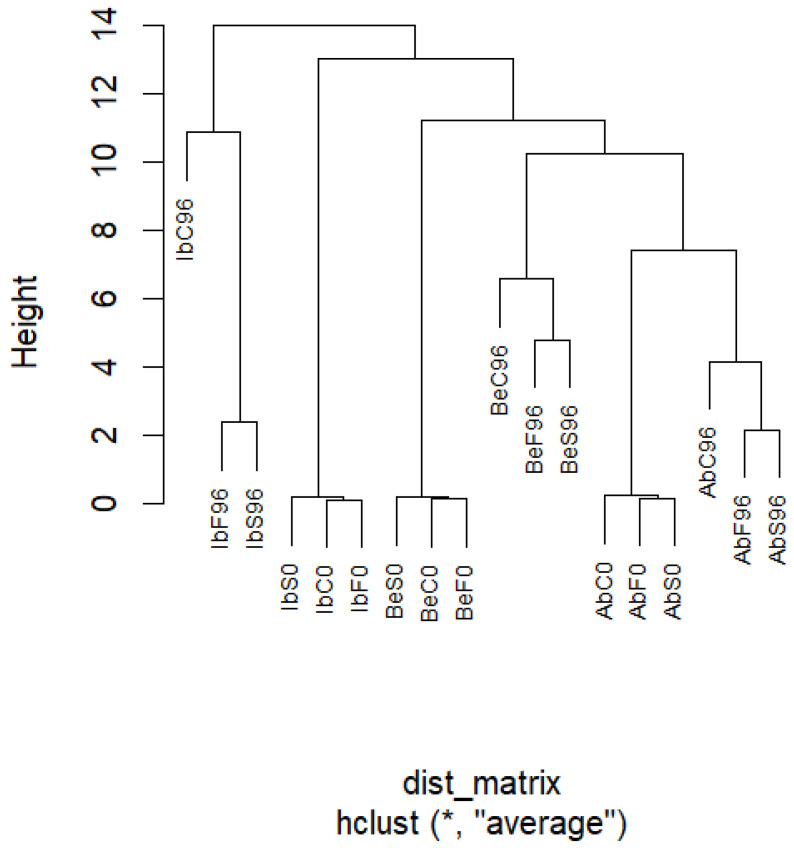
The hierarchical clustering dendrogram of samples.

The proximity of *Agaricus bisporus* samples in time 0 and 96—regardless of the fumigation and distancing of wild mushroom samples in time 0 and 96—indicates that cultivated mushrooms behave more predictably during storage (Figure 7). This might be caused by the different surface microbiomes of wild mushrooms due to uncontrolled growth conditions and a greater diversity of mushrooms revealed during postharvest storage. It is visible that control samples after 96 h of storage differ from fumigated samples in all three fungi species, with *I. badia* being the most glaring. This again supports the notion that changes occurring in wild mushrooms are modified by many factors that are almost impossible to determine. Nevertheless, the cluster analysis highlights that the fumigation of essential oils can effectively modulate postharvest changes.

Summarizing the results from principal component analysis (PCA), hierarchical clustering, and post hoc analysis, it can be stated that both essential oils exhibit comparable effects on mushroom tissues. Both treatments effectively inhibited the increase in free sugars and sugar alcohols across all three species, except for spruce essential oil in *A. bisporus* (for free sugars) and *B. edulis* (for sugar alcohols). However, spruce essential oil demonstrated a more potent inhibitory effect than fennel essential oil in other samples. Thus, while fennel essential oil consistently inhibited these changes, spruce essential oil did not always show this effect, but when it did, the inhibition was more noticeable.

Furthermore, both essential oils effectively inhibited the loss of fatty acids, with fennel essential oil showing the most potent protective effect, particularly in *Imleria badia*, followed by *Agaricus bisporus*. Regarding total free fatty acids, fumigation consistently prevented their accumulation across all species, with *A. bisporus* exhibiting the most pronounced effect, albeit with comparable efficiency across all tested mushrooms.

Finally, the vast majority of vitamins were effectively preserved by fumigation, regardless of mushroom species. It suggests that, under the applied methodology, the overall impact of essential oil fumigation on nutrient retention is mainly independent of the fungi species.

## 3. Materials and Methods

### 3.1. Chemicals and Reagents

Unless stated otherwise, all reagents were purchased from Sigma-Aldrich/Merck (Steinheim, Germany).

### 3.2. Samples and Fumigation Process

Fresh wild mushroom samples (*Boletus edulis* and *Imleria badia*, wild, no cultivar, fully mature, collected from Central Poland mixed forests) and cultivated mushroom samples (*Agaricus bisporus* var. hortensis) were purchased from the local market in September 2023 (Łódź, Central Poland). The samples were immediately transferred to the laboratory, and the species identification of the samples was conducted by a certified mycologist. Essential oils were both 100% pure: fennel (*Foeniculum Vulgare*, derived from seeds, origin: Hungary, cultivar unknown) and spruce (*Picea abies*, derived from needles, origin: Austria, cultivar unknown) were purchased from Aromatherapyoils (Katowice, Poland). The composition of essential oils was analysed using gas chromatography and is available in Supplementary Materials (Tables S7 and S8 and Figures S3 and S4).

Upon arrival, the mushroom fruiting bodies were inspected for potential physical damage and the presence of pests. For the study, mushrooms with comparable colour and weight were selected: approximately 30 g for *B. edulis*, 10 g for *I. badia*, and 20 g for *A. bisporus*. The fruiting bodies were gently cleaned to remove cultivation residues, such as leaves, moss, and peat.

All samples were divided into three sets in three replications, each weighing 500 g. Each mushroom set was placed in a 3 L polypropylene box—9 boxes per fungi species in total. Subsequently, the filter paper (AGF 616, Ø90 mm, Frisenette APS) soaked with 65 µL of each pure essential oil was introduced into the boxes without contact with mushrooms. So, the final concentration was approximately 21.7 µL/L. The boxes were immediately sealed. For the control samples, a dry filter paper was used. After 90 min of exposure, the mushrooms were removed from the treatment boxes and transferred into clean ones. The time of exposure was determined as the time of complete evaporation of essential oils from the filter paper, using the evaporation test procedure [54]. New boxes were sealed with caps, allowing airflow. The whole process, both the fumigation and storage, was at 19 °C and had a relative humidity of 40%. The total storage time was 96 h. This maximum storage time was chosen to exceed the average shelf-life of cultivated mushrooms (1–3 days [3]), after complete decay of the control samples.

Samples were taken from each set at 0 h (prior to treatment), 48 h, and 96 h to perform analysis. Those intended for the vitamin C and vitamins from group B measurements were immediately processed, while the rest of the samples were freeze-dried and stored at −20 °C until the procedures were conducted.

### 3.3. Free Sugar and Free Sugar Alcohol Content

The determination of free sugars followed the method described by Aninowski et al. [55] with modifications. The dried fruiting bodies were ground in a grinder into a fine powder. Then, 2 g samples were placed in extraction thimbles in Soxhlet apparatuses. The samples were extracted using 60 mL of pure for analysis methanol. Then, the solvent was removed by the rotary evaporator. The flasks were left open until the next day for complete dryness. Subsequently, 2 mL of methanol was added and dissolved using an ultrasonic bath until a homogeneous sample was obtained. The extracts were quantitatively transferred to 2 mL glass tightly capped vials. For analysis, 20 μL of each extract was added to chromatographic vials and evaporated to dryness under a gentle stream of nitrogen. Then, 100 µL of pyridine and 100 µL of BSTFA + TMCS (99:1) were added. The standard stock solutions were prepared by dissolving sugars standards in methanol and mixing with pyridine and silylation reagent. The vials were tightly capped and placed on a heating plate at 60 °C for 30 min. After this time, the samples were transferred to microinserts and placed on the tray of a Gerstel MultiPurpose Sampler (MPS 2). GC–MS analyses were performed on a LECO Pegasus 4D device equipped with an Agilent 7890A (Santa Clara, CA, USA) gas chromatograph coupled with a time-of-flight mass spectrometer.

The identification of the compounds was based on standard materials’ retention times and was confirmed by the NIST Spectral Database—the quantification based on the prepared calibration curves for sugar standards. The free sugar and free sugar alcohol concentration was expressed in mg/100 g of dried mushrooms.

### 3.4. Free Fatty Acid and Total Fatty Acid Composition

The analyses were carried out according to the methodology described by Piatek et al. [56] with minor modifications. Briefly, 1 g of dried samples was extracted with 50 mL of hexane in the Soxhlet apparatus. The solvent was removed in a rotary evaporator, leaving the extract overnight. Then, 50 mg of the lipid extract was mixed with 200 µL methyl tert-butyl ether and 200 µL of a 0.25-M solution of trimethylsulfonium hydroxide in methanol. After that, the samples were incubated at 80 °C for 30 min and transformed for the analysis.

The analysis was performed using gas chromatography coupled with time-of-flight mass spectrometry (GC/MS Pegasus 4D, LECO Corp., St. Joseph, MI, USA). The GC column Rt-2560 (100 m × 0.25 mm, 0.20 µm, Restek Corp., Bellefonte, PA, USA, cat. No. 40602) was used. Then, 1 µL of the sample was applied to the split/splitless (SSL) injector in splitless mode (injector temperature 240 °C). The GC oven temperature was initially held at 140 °C for 5 min and then increased to 240 °C for 30 min at a rate of 4 °C/min. Helium as a carrier gas was used at a 1 mL/min flow. Mass spectra were collected using a time-of-flight mass spectrometer. Mass spectrometry settings were as follows: ion source temperature 200 °C, ionisation energy 70 eV, and scan range 33–550 atomic mass units (amu).

The obtained mass spectra were compared with those of NIST/EPA/NIH and Wiley Registry of Mass Spectral Data mass spectral libraries. Retention times of FAMEs were compared to the relative retention times of the standard Supelco 37 Component FAME Mix. The quantification was based on internal standard—nonadecanoic acid (C19:0). The free fatty acid and total fatty acid concentrations were expressed in mg/100 g of dried mushrooms.

### 3.5. Content of α-, β-, γ-, and δ-Tocopherols

The dried fruiting bodies were ground in a grinder into a fine powder. Then, 500 mg of each sample was soaked with 10 mL of hexane and 1 mL of 1% BHT in hexane, and the mixture was shaken at 220 RPM for 1 h and then left overnight. The next day, the suspension was filtered using a Frisenette technical filter paper, and the solvent was evaporated into dryness in a rotary evaporator. Then, 0.5 mL of water was added, and the bulk was placed for 30 min in an ultrasonic bath. The sample was then treated via solid-phase extraction. The SPE column with the C18 phase was conditioned with ultrapure water (HPLC grade). Then, the sample was loaded and passed through the sorbent. The leftovers were solved in methanol, the sample was again loaded onto the SPE column, and the methanol eluate was collected. The leftovers were solved in hexane, and the sample was loaded onto the SPE column to collect the hexane eluate. The column was then washed with hexane, and the eluate was combined with the previous one. Then, the solvent (hexane) was removed using a rotary evaporator, and the bulk was left overnight for complete dryness. The extracts were placed into chromatographic vials with 200 µL of BSTFA+TMCS reagent and underwent silylation at 80 °C for 1 h. After this time, the samples were transferred for analysis.

The analysis was performed using two-dimensional gas chromatography coupled with time-of-flight mass spectrometry (GC/MS Pegasus 4D, LECO Corp., St. Joseph, MI, USA). The following column arrangement was used: the first-dimension column—BPX-5 capillary column (40 m × 0.15 mm i.d., film thickness 0.15 µm)—and the second-dimension column—BPX-50 capillary column (1.8 m × 0.1 mm i.d., film thickness 0.1 µm). The carrier gas was helium at a flow of 1.0 mL/min. The temperature program began at 100 °C for 0.5 min, followed by an increase of 4 °C/min to 310 °C, held for 15 min, and an increase of 10 °C/min to 330 °C, held for 20 min. The total time of the analysis was 90 min. The transfer line temperature was 280 °C, ion-source temperature 200 °C, ionisation energy 70 eV, acquisition rate 150 spectra/s. A two-stage consumable free modulator was cooled to −80 °; temp. was offset rel. to the GC oven: secondary oven +5 °C and modulator +20 °C, respectively. The modulation period was 5 s, hot pulse time 1.5 s, and cool time between stages 1.0 s.

The identification of the compounds was based on standard materials’ retention times and by comparing their mass spectra. The quantification was based on the calibration curves of α-, β-, γ-, and δ-tocopherols. The concentrations were expressed in mg/100 g of dried mushrooms.

### 3.6. Ergocalciferol (Vitamin D2) and Ergosterol Content

The method for fatty acids, ergocalciferol, and ergosterol content analysis was followed by the method for free components analysis described in Cyran et al. [57] with slight modifications. Fresh mushroom samples were cut into smaller parts and dried at 50 °C for 10 h. Then, 2 g of the dried mass was crushed in a mortar and extracted with 50 mL of methanol p.a. in a Soxhlet apparatus. The methanol was removed with a rotary evaporator at 40 °C. The extract was saponified with 10 mL of 2 M NaOH at 40 °C for 2 h. After the extract was cooled down, 4 mL of 6 M HCl was added to the flask, and the hydrolysis of glycosides present in an extract was performed at 100 °C for 1 h under the nitrogen stream. Subsequently, the compounds were extracted three times with diethyl ether. Combined organic phases were dried with anhydrous magnesium sulphate, and the solvent was removed using a rotary evaporator. The samples then underwent silylation with a BSTFA+TMCS reagent (80 °C, 1 h).

The analysis was performed with two-dimensional gas chromatography coupled with time-of-flight mass spectrometry (GC/MS Pegasus 4D, LECO Corp., St. Joseph, MI, USA). The following column arrangement was used: the first-dimension column—BPX-5 capillary column (40 m × 0.15 mm i.d., film thickness 0.15 µm)—the second-dimension column—BPX-50 capillary column (1.8 m × 0.1 mm i.d., film thickness 0.1 µm). The carrier gas was helium at a flow of 0.4 mL/min. The parameters of the mass-spectrometer, modulator, and secondary oven were identical as described in 4.5. The modulation period was 8 s, hot pulse time 2.4 s, and cool time between stages 1.6 s.

The identification of the compounds was based on standard materials’ retention times and by comparing their mass spectra. The quantification was based on the calibration curves of ergosterol and ergocalciferol. The concentrations were expressed in mg/100 g of dried mushrooms.

### 3.7. Content of Ascorbic Acid (Vitamin C)

Vitamin C content in mushrooms was checked using a commercial kit (Ascorbic Acid Assay Kit II, Sigma-Aldrich). Briefly, the concentration of the ascorbic assay was checked via the ferric reducing/antioxidant and ascorbic acid (FRASC) assay. To perform the assay, 10 mg of fresh samples was homogenised with cold FRASC buffer and centrifuged at 13,0000× *g* for 10 min at 4 °C following the assay reaction. The absorbance was measured at 593 nm on a Multiscan GO Microplate Spectrophotometer (Thermo Scientific, Waltham, MA, USA). The ascorbic acid standard was used for the quantitative analysis to prepare the standard curve. The concentrations were expressed in mg/100 g of fresh mushrooms.

### 3.8. Content of Vitamins B_1_, B_2_, and B_6_

The analysis of vitamins B_1_, B_2_, and B_6_ was performed based on the method described in [58] with modifications. The raw fruiting bodies of mushrooms were thoroughly homogenised, and then 3 g of the sample was mixed with 30 mL of 0.1 N hydrochloric acid. The mixture was then heated at 100 °C for 25 min. Then, the samples were cooled to 30 °C, and the pH was adjusted to 4.0 using 5 M sodium acetate. After that, 500 mg of takadiastase from *Aspergillus oryzae* was added to each sample, and the mixtures were heated to 55 °C for 3 h, mixing with a magnetic stirrer to perform the hydrolysis. The samples were cooled down, 1 mL of 50% trichloroacetic acid was added, and the samples were again heated to 100 °C for 5 min. Then, 20 mL of 0.1 N HCl was added to the cooled samples, and the extract was filtered using a No.1 Whatman filter paper. Then, 300 µL of 1% potassium ferricyanide in 15% NaOH solution was added to 5 mL of the filtrated, and the samples were left to react in the dark for 10 min. The extracts were neutralised with 200 µL of 15% orthophosphoric acid and filtered using a syringe filter of 0.22 µL. The ready samples were immediately transferred to the chromatography analysis.

The analysis was performed with high-performance liquid chromatography (Dionex STH 585 Column Oven, SpectraLab Scientific Inc., Markham, ON, Canada) coupled with a UV–VIS/DAD detector (Dionex, UVD340S). The mobile phase consisted of potassium phosphate buffer (0.01 M, pH 7), and the organic phase consisted of methanol. Initially, the mobile phase consisted of a 100% aqueous phase, with a 0.9 mL/min flow rate, and was held for 1 min. Then, at 1.01, the linear gradient arriving at 90% of the aqueous phase and 10% in 4 min was introduced and followed by the linear gradient arriving at 50% of the aqueous phase and 50% of the organic phase in 15 min, with a flow rate of 1.5 mL/min. The HPLC column XTerra RP18 (Waters), 5 µm, 4.6 × 150 mm was used, and the oven temperature was set at 25 °C. The vitamins were monitored at wavelengths of λ_B6_ = 221 nm, λ_B1_ = 238 nm, and λ_B2_ = 267 nm. The identification of the compounds was based on standard materials’ retention times and by comparing their UV spectra. The quantification was based on the calibration curves of vitamins B_1_, B_2_, and B_6_ standard solutions. The concentrations were expressed in mg/100 g of fresh mushrooms.

### 3.9. Statistical Analysis

Statistical analyses were conducted using RStudio with R version 4.4.2. The dataset comprised measurements at different time points: two-time points (0 h and 96 h) for fatty acids and free fatty acid analysis and three-time points (0 h, 48 h, and 96 h) for other compounds.

A linear model incorporating three-way interactions (Variant × Time × Fungus) was constructed using the stats package. Prior to analysis, normality and homogeneity of variance assumptions were verified using Shapiro–Wilk and Levene’s tests, respectively. The dependent variable underwent a logarithmic transformation. The model included all possible two-way and three-way interactions, yielding *t*-values and *p*-values. Type III ANOVA was performed using the car package to assess main effects and interactions, providing F-statistics and *p*-values. Post hoc analyses were conducted using the emmeans package, with Tukey’s test generating *p*-value matrices for pairwise comparisons. The estimated marginal means and confidence intervals were back-transformed to the original scale. Statistical grouping was determined using the multcompView algorithm.

Principal component analysis (PCA) was performed on Z-score standardised values for individual compounds (or compound groups when applicable) using the prcomp function. The analysis included an examination of variance explained by principal components, loading matrices for feature-level analysis, and sample distribution patterns. Feature-level analysis was conducted in two dimensions (PC1 and PC2), while sample distribution was analysed in three dimensions (PC1, PC2, and PC3). Component rotation values were evaluated to determine feature contributions.

Hierarchical cluster analysis was performed using a distance matrix based on the first five principal components (PC1-PC5). The analysis employed the Manhattan distance metric and the UPGMA (Unweighted Pair Group Method with Arithmetic Mean) clustering method, also known as the average linkage method.

### 3.10. Visualisation

All visualisations were generated using R version 4.4.2 in RStudio. Bar plots and two-dimensional PCA plots were created using the ggplot2 package with colour schemes from RColorBrewer. Three-dimensional PCA visualisations were generated using the plotly package in conjunction with RColorBrewer. The hierarchical clustering dendrogram was created using the hclust function from the stats package.

## 4. Conclusions

This study demonstrated the potential of essential oil fumigation as an effective treatment for prolonging the shelf-life of wild and cultivated mushrooms by delaying the loss of most key nutrients. The results have shown that fennel and spruce essential oils applied in vapours can enhance the protective mechanisms of mushrooms and inhibit undesirable changes, such as polysaccharide or lipid breakdowns. As a result, after 4 days of storage in non-optimal conditions, fumigated samples suffered a minor increase in free fatty acids, free sugars, and free alcohols. Moreover, vapour treatment preserved the content of unsaturated fatty acids (434–443 mg/100 g in fumigated samples in comparison to 339 mg/100 g control samples for *B. edulis* after storage) and vitamin C (9.50–11 mg/100 g in fumigated samples in comparison to 5.50 mg/100 g control samples for *A. bisporus*) and even caused a slight increase in ergocalciferol content (from 1.25 to 9.52 µg/100 g in *I. badia* fumigated with spruce essential oil after storage). Several capabilities of essential oils might cause such results. First of all, compounds present in those mixtures may cause mild metabolic stress that triggers the signalling pathways to activate defence mechanisms that stimulate tissues to increase primary and secondary antioxidants [6,18,19,20,52]. Also, essential oils are known for their antimicrobial properties; hence, the inhibition of microbial growth can decrease the oxidative stress in mushroom tissues and diminish the losses of nutrients occurring due to microbial metabolism [4,5]. Hence, the effectiveness of the fumigation of essential oils in preserving the nutrients in mushrooms is why the topic should be explored in more depth.

Further research should aim to explore the underlying mechanisms responsible for the observed preservation effects, particularly the antioxidant response and microbial inhibition pathways. It would also be valuable to expand the analysis to other mushroom species, additional essential oils, and their combinations to assess potential synergistic effects. What is more, future studies should evaluate the long-term safety and potential residues of essential oils in food products, particularly when used in commercial settings. Comparative assessments with conventional preservation techniques could help define the advantages and limitations of essential oil fumigation more precisely. Finally, long-term storage trials under various environmental conditions should be considered to better reflect different storage and distribution systems.

## Figures and Tables

**Table 1:** The average content of free sugars (mg/100 g d.w.) in *B. edulis*, *I. badia*, and *A. bisporus* before the treatment and storage (0 h) and after 96 h of storage.

Sugar		*Boletus edulis*		*Imleria badia*		*Agaricus bisporus*	
		0 h	96 h	0 h	96 h	0 h	96 h
Glucose	Control	52.6 ± 3.98 ^a^	123.07 ± 22.92 ^c^	10.49 ± 0.84 ^ab^	16.92 ± 1.41 ^c^	37.71 ± 1.56 ^a^	134.21 ± 6.99 ^b^
	Fennel	49.16 ± 2.39 ^a^	68.58 ± 5.03 ^b^	11.09 ± 0.99 ^ab^	12.83 ± 1.46 ^b^	36.89 ± 1.11 ^a^	119.68 ± 5.73 ^bc^
	Spruce	57.02 ± 5.13 ^ab^	135.57 ± 8.06 ^c^	9.96 ± 1.40 ^a^	8.89 ± 0.80 ^a^	38.21 ± 1.60 ^a^	108.86 ± 5.31 ^c^
Fructose	Control	0.16 ± 0.03 ^a^	4.02 ± 0.35 ^b^	7.57 ± 0.86 ^a^	10.56 ± 0.77 ^b^	0.10 ± 0.01 ^a^	1.10 ± 0.13 ^b^
	Fennel	0.16 ± 0.01 ^a^	7.29 ± 0.34 ^c^	7.25 ± 0.49 ^a^	2.38 ± 0.29 ^c^	0.09 ± 0.01 ^a^	0.07 ± 0.02 ^a^
	Spruce	0.17 ± 0.01 ^a^	2.58 ± 0.19 ^d^	7.31 ± 0.43 ^a^	4.45 ± 0.39 ^d^	0.10 ± 0.01 ^a^	0.09 ± 0.01 ^a^
Xylose	Control	15.21 ± 1.05 ^a^	49.46 ± 3.68 ^b^	10.58 ± 0.67 ^a^	47.88 ± 2.59 ^b^	0.33 ± 0.04 ^a^	3.43 ± 0.75 ^bc^
	Fennel	16.25 ± 0.92 ^a^	54.35 ± 2.97 ^b^	10.61 ± 0.77 ^a^	11.67 ± 1.37 ^a^	0.33 ± 0.06 ^a^	4.01 ± 0.20 ^b^
	Spruce	15.63 ± 1.10 ^a^	27.31 ± 2.61 ^c^	10.62 ± 0.67 ^a^	34.68 ± 3.08 ^c^	0.34 ± 0.05 ^a^	2.80 ± 0.15 ^c^
Trehalose	Control	106.14 ± 5.92 ^ab^	721.57 ± 11.86 ^c^	557.85 ± 15.47 ^a^	813.14 ± 25.41 ^b^	177.06 ± 3.27 ^a^	287.04 ± 20.58 ^b^
	Fennel	114.08 ± 5.54 ^ab^	650.52 ± 18.62 ^c^	555.04 ± 23.37 ^a^	756.32 ± 28.02 ^b^	174.77 ± 5.65 ^a^	216.43 ± 8.69 ^c^
	Spruce	117.31 ± 7.57 ^a^	102.54 ± 6.12 ^b^	556.67 ± 19.16 ^a^	429.24 ± 25.05 ^c^	173.64 ± 4.26 ^a^	290.37 ± 11.71 ^b^
Sucrose	Control	*nd*	19.92 ± 3.24 ^a^	0.68 ± 0.06 ^a^	51.03 ± 3.69 ^b^	*nd*	1.38 ± 0.15 ^a^
	Fennel	*nd*	32.49 ± 3.61 ^b^	0.67 ± 0.07 ^a^	15.37 ± 2.44 ^c^	*nd*	0.82 ± 0.07 ^b^
	Spruce	*nd*	18.01 ± 1.49 ^a^	0.69 ± 0.07 ^a^	17.07 ± 2.51 ^c^	*nd*	0.88 ± 0.13 ^b^
Total sugars	Control	174.11 ± 7.95 ^a^	918.05 ± 19.78 ^c^	587.16 ± 17.67 ^a^	939.51 ± 29.38 ^b^	215.20 ± 2.67 ^a^	425.79 ± 27.53 ^b^
	Fennel	179.65 ± 6.84 ^ab^	813.24 ± 23.64 ^d^	584.66 ± 21.84 ^a^	798.57 ± 32.46 ^c^	212.09 ± 6.45 ^a^	340.27 ± 11.77 ^c^
	Spruce	190.13 ± 7.10 ^b^	286.01 ± 1.05 ^e^	585.25 ± 19.69 ^a^	494.34 ± 19.99 ^d^	212.29 ± 5.74 ^a^	402.11 ± 10.67 ^b^

Values are means of three replicates ± standard deviation. Different lowercase letters (^a^, ^b^, ^c^, ^d^ and ^e^) indicate statistically significant differences (*p* ≤ 0.05) between all treatment variants and storage times within the same compound and mushroom species, according to Tukey’s HSD test. Abbreviation: *nd*—not detected.

**Table 2:** The average content of free sugar alcohols (mg/100 g d.w.) in *B. edulis*, *I. badia* and *A. bisporus* before the treatment and storage (0 h) and after 96 h of storage.

Sugar Alcohol		*Boletus edulis*		*Imleria badia*		*Agaricus bisporus*	
		0 h	96 h	0 h	96 h	0 h	96 h
Sorbitol	Control	6.06 ± 0.08 ^a^	15.49 ± 1.85 ^b^	*nd*	*nd*	15.48 ± 0.52 ^a^	98.72 ± 8.66 ^b^
	Fennel	6.02 ± 0.05 ^a^	41.94 ± 4.54 ^c^	*nd*	*nd*	17.28 ± 0.53 ^a^	85.84 ± 3.65 ^b^
	Spruce	6.12 ± 0.06 ^a^	22.50 ± 3.02 ^d^	*nd*	*nd*	15.73 ± 0.71 ^a^	27.98 ± 7.64 ^c^
Mannitol	Control	33.73 ± 1.57 ^a^	238.32 ± 7.99 ^b^	222.14 ± 6.96 ^a^	974.01 ± 65.50 ^b^	86.38 ± 3.50 ^a^	258.03 ± 6.28 ^b^
	Fennel	33.45 ± 3.49 ^a^	295.38 ± 7.64 ^c^	222.37 ± 7.00 ^a^	745.50 ± 23.63 ^c^	85.68 ± 4.00 ^a^	322.25 ± 15.83 ^c^
	Spruce	34.19 ± 2.57 ^a^	221.30 ± 10.41 ^b^	224.10 ± 5.88 ^a^	613.60 ± 14.24 ^d^	85.55 ± 3.44 ^a^	225.43 ± 15.01 ^d^
Arabitol	Control	2.41 ± 0.18 ^a^	26.77 ± 4.49 ^b^	20.76 ± 1.06 ^a^	147.97 ± 9.17 ^b^	1.86 ± 0.12 ^a^	4.5 ± 0.27 ^b^
	Fennel	2.77 ± 0.03 ^a^	13.28 ± 2.00 ^c^	20.41 ± 0.86 ^a^	120.57 ± 10.06 ^c^	1.80 ± 0.16 ^a^	3.23 ± 0.38 ^c^
	Spruce	2.69 ± 0.08 ^a^	13.45 ± 1.92 ^c^	20.37 ± 0.61 ^a^	146.27 ± 7.04 ^b^	1.85 ± 0.17 ^a^	2.72 ± 0.19 ^c^
Trehalose	Control	*nd*	3.84 ± 0.27 ^a^	0.39 ± 0.16 ^a^	145.96 ± 12.35 ^b^	3.16 ± 0.09 ^a^	13.74 ± 2.51 ^bc^
	Fennel	*nd*	3.80 ± 0.27 ^a^	0.41 ± 0.19 ^a^	126.85 ± 8.45 ^b^	3.22 ± 0.08 ^a^	14.89 ± 1.74 ^b^
	Spruce	*nd*	3.02 ± 0.20 ^a^	0.43 ± 0.15 ^a^	141.64 ± 7.51 ^b^	3.24 ± 0.05 ^a^	10.99 ± 1.87 ^c^
Total sugar alcohols	Control	42.20 ± 1.43 ^a^	284.42 ± 12.70 ^b^	243.29 ± 7.04 ^a^	1267.94 ± 86.83 ^b^	106.88 ± 3.94 ^a^	375.00 ± 12.05 ^b^
	Fennel	42.24 ± 3.56 ^a^	354.39 ± 8.24 ^c^	243.19 ± 6.08 ^a^	992.92 ± 5.95 ^c^	107.98 ± 4.61 ^a^	426.21 ± 17.41 ^c^
	Spruce	43.00 ± 2.37 ^a^	260.27 ± 11.85 ^b^	244.90 ± 5.82 ^a^	901.50 ± 28.21 ^c^	106.37 ± 4.01 ^a^	267.13 ± 9.42 ^d^

Values are means of three replicates ± standard deviation. Different lowercase letters (^a^, ^b^, ^c^, and ^d^) indicate statistically significant differences (*p* ≤ 0.05) between all treatment variants and storage times within the same compound and mushroom species, according to Tukey’s HSD test. Abbreviation: *nd*—not detected.

**Table 3:** Average content (mg/100 g d.w.) and percentage composition of fatty acids (%) in *B. edulis* before the treatment and storage (0 h) and after 96 h of storage.

	*Boletus edulis*									
	0 h				96 h					
	Control	Fennel	Spruce	%	Control	%	Fennel	%	Spruce	%
Lauric Acid (C12:0)	0.79 ± 0.08 ^a^	0.70 ± 0.08 ^a^	0.71 ± 0.10 ^a^	0.06 ± 0.01	0.25 ± 0.02 ^b^	0.05 ± 0.00	0.38 ± 0.03 ^c^	0.06 ± 0.01	0.17 ± 0.08 ^d^	0.03 ± 0.02
Myristic Acid (C14:0)	2.35 ± 0.07 ^a^	2.42 ± 0.12 ^a^	2.42 ± 0.17 ^a^	0.21 ± 0.02	2.11 ± 0.15 ^a^	0.43 ± 0.04	0.93 ± 0.16 ^b^	0.15 ± 0.03	0.99 ± 0.21 ^b^	0.16 ± 0.03
Pentadecanoic Acid (C15:0)	2.90 ± 0.28 ^a^	2.89 ± 0.41 ^a^	2.81 ± 0.46 ^a^	0.25 ± 0.04	1.98 ± 0.12 ^b^	0.40 ± 0.02	2.52 ± 0.12 ^ab^	0.41 ± 0.02	2.81 ± 0.83 ^a^	0.47 ± 0.03
Palmitic Acid (C16:0)	117.57 ± 6.70 ^a^	116.50 ± 5.03 ^a^	116.97 ± 7.67 ^a^	10.38 ± 0.67	99.74 ± 2.06 ^b^	20.28 ± 0.82	113.42 ± 2.85 ^a^	18.52 ± 0.66	109.14 ± 6.61 ^ab^	18.08 ± 0.11
Palmitoleic Acid (C16:1 n-7c)	11.01 ± 1.64 ^a^	12.27 ± 1.46 ^a^	11.55 ± 0.84 ^a^	1.03 ± 0.08	7.55 ± 0.36 ^b^	1.54 ± 0.10	5.51 ± 0.28 ^c^	0.90 ± 0.06	6.37 ± 0.42 ^bc^	1.05 ± 0.07
Hexadecadienoic Acid (C16:2 n-6c,9c)	3.82 ± 0.22 ^a^	3.78 ± 0.11 ^a^	3.75 ± 0.15 ^a^	0.33 ± 0.01	2.09 ± 0.13 ^b^	0.42 ± 0.02	1.87 ± 0.23 ^b^	0.30 ± 0.05	0.20 ± 0.04 ^c^	0.03 ± 0.01
Margaric Acid (C17:0)	11.87 ± 1.27 ^a^	11.59 ± 1.07 ^a^	12.37 ± 1.11 ^a^	1.10 ± 0.10	8.53 ± 0.44 ^b^	1.73 ± 0.06	4.50 ± 0.18 ^c^	0.74 ± 0.04	7.11 ± 0.10 ^d^	1.18 ± 0.03
Heptadecenoic Acid (C17:1 n-7c)	1.28 ± 0.17 ^a^	1.41 ± 0.23 ^a^	1.26 ± 0.13 ^a^	0.11 ± 0.01	*nd*	—	*nd*	—	*nd*	—
Stearic Acid (C18:0)	34.80 ± 3.72 ^a^	35.50 ± 2.21 ^a^	36.54 ± 1.86 ^a^	3.24 ± 0.18	27.88 ± 3.18 ^b^	5.67 ± 0.62	34.25 ± 2.74 ^a^	5.59 ± 0.58	34.08 ± 8.99 ^a^	5.65 ± 0.35
Oleic Acid (C18:1 n-9c)	330.00 ± 17.02 ^a^	333.22 ± 15.94 ^a^	326.48 ± 13.94 ^a^	28.97 ± 1.26	136.00 ± 9.47 ^b^	27.66 ± 2.21	189.41 ± 9.64 ^c^	30.92 ± 1.01	190.33 ± 9.65 ^c^	31.52 ± 1.05
Linoleic Acid (C18:2 n-6c,9c)	549.88 ± 13.74 ^a^	552.48 ± 15.27 ^a^	566.02 ± 12.75 ^a^	50.23 ± 0.94	187.33 ± 16.33 ^b^	38.10 ± 2.38	240.05 ± 13.94 ^c^	39.19 ± 1.40	299.74 ± 12.25 ^c^	38.05 ± 1.12
α-Linolenic Acid (C18:3 n-3c,6c,9c)	4.81 ± 0.46 ^a^	4.59 ± 0.36 ^a^	4.66 ± 0.24 ^a^	0.41 ± 0.02	0.94 ± 0.12 ^b^	0.19 ± 0.03	1.67 ± 0.24 ^c^	0.27 ± 0.05	2.64 ± 0.32 ^d^	0.44 ± 0.04
Arachidic Acid (C20:0)	19.51 ± 2.42 ^a^	21.93 ± 2.24 ^a^	18.97 ± 1.17 ^a^	1.68 ± 0.11	8.85 ± 2.31 ^b^	1.80 ± 0.44	9.14 ± 0.46 ^bc^	1.49 ± 0.04	12.18 ± 0.88 ^c^	2.02 ± 0.13
Gondoic Acid (C20:1 n-9c)	14.09 ± 0.75 ^a^	14.99 ± 0.69 ^a^	13.97 ± 0.54 ^a^	1.24 ± 0.04	6.32 ± 0.34 ^b^	1.29 ± 0.07	4.86 ± 0.19 ^c^	0.79 ± 0.05	4.30 ± 0.36 ^c^	0.71 ± 0.04
Eicosadienoic Acid (C20:2 n-6c,11c)	1.71 ± 0.07 ^a^	1.68 ± 0.15 ^a^	1.83 ± 0.16 ^a^	0.16 ± 0.01	0.10 ± 0.02 ^b^	0.02 ± 0.00	0.84 ± 0.06 ^c^	0.14 ± 0.01	0.14 ± 0.10 ^c^	0.07 ± 0.04
Heneicosanoic Acid (C21:0)	1.93 ± 0.46 ^a^	1.61 ± 0.12 ^a^	1.71 ± 0.12 ^a^	0.15 ± 0.01	0.20 ± 0.05 ^b^	0.04 ± 0.01	0.65 ± 0.13 ^c^	0.11 ± 0.02	0.11 ± 0.10 ^c^	0.10 ± 0.02
Behenic Acid (C22:0)	3.99 ± 0.65 ^a^	3.52 ± 0.36 ^ab^	3.47 ± 0.24 ^ab^	0.31 ± 0.02	1.85 ± 0.20 ^c^	0.38 ± 0.03	2.53 ± 0.33 ^cd^	0.41 ± 0.07	2.73 ± 0.35 ^bd^	0.45 ± 0.05
Docosadienoic Acid (C22:2 n-6c,13c)	1.46 ± 0.13 ^a^	1.48 ± 0.15 ^a^	1.42 ± 0.10 ^a^	0.13 ± 0.01	*nd*	—	*nd*	—	0.42 ± 0.04 ^b^	0.07 ± 0.00
SFA	195.69 ± 6.50 ^a^	196.67 ± 10.01 ^a^	195.97 ± 4.18 ^a^	17.39 ± 0.37	151.38 ± 2.56 ^b^	30.79 ± 0.47	168.33 ± 2.23 ^c^	27.48 ± 1.11	169.77 ± 10.27 ^c^	28.12 ± 0.38
MUFA	356.39 ± 17.16 ^a^	361.90 ± 14.01 ^a^	353.27 ± 13.67 ^a^	31.35 ± 1.25	149.88 ± 9.45 ^b^	30.48 ± 2.25	199.78 ± 9.50 ^c^	32.61 ± 1.01	201.00 ± 9.92 ^c^	33.29 ± 1.07
PUFA	561.69 ± 14.09 ^a^	564.00 ± 15.03 ^a^	577.69 ± 12.71 ^a^	51.26 ± 0.94	190.45 ± 16.37 ^b^	38.73 ± 2.38	244.43 ± 13.67 ^c^	39.90 ± 1.37	233.01 ± 12.41 ^c^	38.59 ± 1.09
Total	1113.77 ± 11.07 ^a^	1122.57 ± 7.59 ^a^	1126.92 ± 7.16 ^a^		491.71 ± 14.56 ^b^		612.54 ± 17.25 ^c^		603.78 ± 28.48 ^c^	
UFA/SFA	4.69 ± 0.13 ^a^	4.72 ± 0.25 ^a^	4.75 ± 0.12 ^a^		2.25 ± 0.05 ^b^		2.64 ± 0.15 ^c^		2.56 ± 0.05 ^c^	

Values are means of three replicates ± standard deviation. Different lowercase letters (^a^, ^b^, ^c^, and ^d^) indicate statistically significant differences (*p* ≤ 0.05) between all treatment variants and storage times within the same compound and mushroom species, according to Tukey’s HSD test. Abbreviation: *nd*—not detected. SFA—saturated fatty acids; MUFA—monounsaturated fatty acids; PUFA—polyunsaturated fatty acids; UFA/SFA—ratio between unsaturated and saturated fatty acids.

**Table 4:** Average content (mg/100 g d.w.) and percentage composition of fatty acids (%) in *I. badia* before the treatment and storage (0 h) and after 96 h of storage.

	*Imleria badia*									
	0 h				96 h					
	Control	Fennel	Spruce	%	Control	%	Fennel	%	Spruce	%
Lauric Acid (C12:0)	0.93 ± 0.06 ^a^	0.89 ± 0.08 ^a^	0.92 ± 0.11 ^a^	0.07 ± 0.01	0.61 ± 0.06 ^b^	0.23 ± 0.03	0.17 ± 0.02 ^c^	0.02 ± 0.00	0.59 ± 0.03 ^b^	0.07 ± 0.00
Myristic Acid (C14:0)	3.18 ± 0.13 ^a^	3.19 ± 0.24 ^a^	3.39 ± 0.41 ^a^	0.23 ± 0.02	1.73 ± 0.24 ^b^	0.76 ± 0.10	1.23 ± 0.15 ^c^	0.12 ± 0.01	0.92 ± 0.04 ^d^	0.10 ± 0.01
Pentadecanoic Acid (C15:0)	10.52 ± 1.38 ^a^	10.48 ± 1.01 ^a^	10.34 ± 0.41 ^a^	0.76 ± 0.06	5.71 ± 0.85 ^b^	19.47 ± 0.52	4.84 ± 1.01 ^b^	0.48 ± 0.10	8.98 ± 0.12 ^a^	0.99 ± 0.02
Palmitic Acid (C16:0)	225.86 ± 13.03 ^a^	220.33 ± 8.97 ^a^	221.44 ± 9.96 ^a^	16.29 ± 0.52	146.24 ± 6.70 ^b^	0.27 ± 0.03	178.86 ± 9.12 ^a^	17.89 ± 0.85	171.20 ± 7.02 ^ab^	18.80 ± 0.59
Palmitoleic Acid (C16:1 n-7c)	4.56 ± 0.17 ^a^	4.43 ± 0.32 ^a^	4.36 ± 0.33 ^a^	0.33 ± 0.03	2.03 ± 0.21 ^b^	0.06 ± 0.00	0.80 ± 0.06 ^c^	0.08 ± 0.01	3.13 ± 0.12 ^d^	0.34 ± 0.01
Margaric Acid (C17:0)	1.65 ± 0.13 ^a^	1.72 ± 0.17 ^a^	1.66 ± 0.14 ^a^	0.12 ± 0.01	0.47 ± 0.03 ^b^	8.94 ± 0.43	1.03 ± 0.10 ^c^	0.10 ± 0.01	1.61 ± 0.09 ^a^	0.18 ± 0.01
Stearic Acid (C18:0)	76.60 ± 7.97 ^ab^	77.20 ± 8.97 ^a^	79.31 ± 7.58 ^a^	5.53 ± 0.65	67.14 ± 3.71 ^a^	19.70 ± 0.88	74.37 ± 4.31 ^a^	7.44 ± 0.44	69.09 ± 0.78 ^a^	7.59 ± 0.04
Oleic Acid (C18:1 n-9c)	229.24 ± 13.03 ^a^	225.51 ± 9.40 ^a^	230.73 ± 13.40 ^a^	16.54 ± 0.78	147.97 ± 3.94 ^b^	49.85 ± 0.54	190.48 ± 7.84 ^c^	19.06 ± 0.74	173.03 ± 5.81 ^c^	19.00 ± 0.48
Linoleic Acid (C18:2 n-6c,9c)	812.75 ± 17.25 ^a^	816.51 ± 18.98 ^a^	814.06 ± 21.63 ^a^	58.63 ± 1.22	374.36 ± 0.85 ^b^	0.01 ± 0.01	537.50 ± 13.19 ^c^	53.77 ± 1.46	474.70 ± 9.92 ^d^	52.13 ± 1.06
α-Linolenic Acid (C18:3 n-3c,6c,9c)	1.33 ± 0.07 ^a^	1.36 ± 0.06 ^a^	1.33 ± 0.06 ^a^	0.10 ± 0.00	0.10 ± 0.06 ^b^	0.23 ± 0.03	1.04 ± 0.07 ^c^	0.10 ± 0.01	0.02 ± 0.01 ^b^	0.00 ± 0.00
Gondoic Acid (C20:1 n-9c)	4.92 ± 0.08 ^a^	4.91 ± 0.11 ^a^	4.91 ± 0.11 ^a^	0.35 ± 0.00	*nd*	—	0.85 ± 0.08 ^b^	0.09 ± 0.01	0.02 ± 0.01 ^c^	0.00 ± 0.00
Eicosadienoic Acid (C20:2 n-6c,11c)	5.43 ± 0.24 ^a^	5.26 ± 0.95 ^a^	5.21 ± 0.41 ^a^	0.39 ± 0.03	*nd*	—	0.71 ± 0.03 ^b^	0.07 ± 0.00	*nd*	—
Heneicosanoic Acid (C21:0)	1.44 ± 0.11 ^a^	1.43 ± 0.05 ^a^	1.40 ± 0.05 ^a^	0.10 ± 0.00	0.27 ± 0.04 ^b^	0.04 ± 0.01	1.36 ± 0.17 ^c^	0.14 ± 0.02	1.09 ± 0.09 ^c^	0.12 ± 0.01
Behenic Acid (C22:0)	7.22 ± 1.01 ^a^	6.48 ± 1.01 ^a^	6.84 ± 0.94 ^a^	0.52 ± 0.08	4.43 ± 0.25 ^b^	0.59 ± 0.04	5.92 ± 0.29 ^a^	0.59 ± 0.03	6.20 ± 0.26 ^a^	0.68 ± 0.03
Erucic Acid (C22:1 n-9c)	0.18 ± 0.01 ^a^	0.18 ± 0.01 ^a^	0.19 ± 0.02 ^a^	0.01 ± 0.00	*nd*	—	0.16 ± 0.02 ^a^	0.02 ± 0.00	*nd*	—
Nervonic Acid (C24:1 n-9c)	0.37 ± 0.05 ^a^	0.40 ± 0.03 ^a^	0.37 ± 0.03 ^a^	0.03 ± 0.00	*nd*	—	0.23 ± 0.02 ^b^	0.02 ± 0.00	*nd*	—
SFA	327.41 ± 14.07 ^a^	321.73 ± 13.81 ^a^	325.30 ± 9.67 ^a^	23.62 ± 1.03	226.60 ± 9.22 ^b^	30.17 ± 0.64	267.78 ± 8.26 ^c^	26.79 ± 0.74	259.68 ± 7.95 ^c^	28.52 ± 0.60
MUFA	239.26 ± 9.79 ^a^	235.43 ± 9.78 ^a^	240.57 ± 13.35 ^a^	17.26 ± 0.81	150.00 ± 3.95 ^b^	19.97 ± 0.89	192.52 ± 7.89 ^c^	19.26 ± 0.75	176.17 ± 5.71 ^c^	19.35 ± 0.46
PUFA	819.51 ± 20.87 ^a^	823.13 ± 19.25 ^a^	820.60 ± 21.29 ^a^	59.12 ± 1.20	374.43 ± 9.87 ^b^	49.86 ± 0.54	539.24 ± 13.19 ^c^	53.95 ± 1.46	474.71 ± 9.91 ^d^	52.13 ± 1.06
Total	1386.18 ± 8.83 ^a^	1380.30 ± 12.91 ^a^	1386.47 ± 15.06 ^a^		751.02 ± 14.89 ^b^		999.54 ± 3.37 ^c^		910.57 ± 14.09 ^d^	
UFA/SFA	3.24 ± 0.18 ^a^	3.29 ± 0.16 ^a^	3.26 ± 0.14 ^a^		2.32 ± 0.07 ^b^		2.73 ± 0.10 ^c^		2.51 ± 0.07 ^bc^	

Values are means of three replicates ± standard deviation. Different lowercase letters (^a^, ^b^, ^c^, and ^d^) indicate statistically significant differences (*p* ≤ 0.05) between all treatment variants and storage times within the same compound and mushroom species, according to Tukey’s HSD test. Abbreviation: *nd*—not detected. SFA—saturated fatty acids; MUFA—monounsaturated fatty acids; PUFA—polyunsaturated fatty acids; UFA/SFA—ratio between unsaturated and saturated fatty acids.

**Table 5:** Average content (mg/100 g d.w.) percentage composition of fatty acids (%) in *A. bisporus* before the treatment and storage (0 h) and after 96 h of storage.

	*Agaricus bisporus*									
	0 h				96 h					
	Control	Fennel	Spruce	%	Control	%	Fennel	%	Spruce	%
Lauric Acid (C12:0)	0.87 ± 0.06 ^a^	0.91 ± 0.03 ^a^	0.86 ± 0.10 ^a^	0.08 ± 0.01	0.63 ± 0.04 ^b^	0.12 ± 0.01	0.55 ± 0.03 ^bc^	0.07 ± 0.00	0.43 ± 0.04 ^c^	0.06 ± 0.01
Myristic Acid (C14:0)	9.42 ± 0.13 ^ab^	9.77 ± 0.15 ^a^	9.14 ± 0.15 ^a^	0.91 ± 0.03	6.84 ± 0.50 ^b^	1.27 ± 0.13	6.31 ± 0.29 ^bc^	0.86 ± 0.05	5.73 ± 0.24 ^c^	0.87 ± 0.03
Pentadecanoic Acid (C15:0)	23.16 ± 1.38 ^a^	22.31 ± 0.30 ^a^	24.01 ± 0.42 ^b^	2.23 ± 0.12	17.31 ± 1.50 ^b^	3.20 ± 0.34	18.38 ± 1.07 ^b^	2.50 ± 0.12	19.43 ± 1.33 ^a^	2.94 ± 0.13
Palmitic Acid (C16:0)	117.51 ± 13.03 ^a^	112.20 ± 3.14 ^a^	111.03 ± 3.10 ^a^	11.31 ± 0.28	89.23 ± 3.38 ^b^	16.51 ± 0.90	97.94 ± 3.40 ^b^	13.31 ± 0.19	95.92 ± 1.92 ^b^	14.54 ± 0.60
Palmitoleic Acid (C16:1 n-7c)	3.36 ± 0.33 ^ab^	3.94 ± 0.09 ^a^	4.04 ± 0.10 ^a^	0.32 ± 0.04	1.23 ± 0.44 ^b^	0.23 ± 0.08	2.41 ± 0.28 ^c^	0.33 ± 0.04	3.52 ± 0.18 ^a^	0.53 ± 0.04
Margaric Acid (C17:0)	8.13 ± 0.50 ^a^	8.97 ± 0.19 ^a^	9.02 ± 0.24 ^a^	0.78 ± 0.05	6.05 ± 0.52 ^b^	1.12 ± 0.11	6.69 ± 0.41 ^c^	0.91 ± 0.07	8.23 ± 0.32 ^a^	1.25 ± 0.06
Stearic Acid (C18:0)	69.44 ± 4.53 ^a^	70.35 ± 3.46 ^a^	63.41 ± 0.10 ^ab^	6.68 ± 0.34	49.33 ± 4.05 ^c^	9.12 ± 0.50	56.22 ± 2.96 ^bc^	7.64 ± 0.40	50.06 ± 2.20 ^c^	7.59 ± 0.09
Oleic Acid (C18:1 n-9c)	208.92 ± 12.90 ^a^	195.82 ± 6.15 ^a^	186.88 ± 0.35 ^a^	20.11 ± 1.07	106.42 ± 6.06 ^b^	19.69 ± 1.06	134.48 ± 6.45 ^c^	18.28 ± 0.84	122.68 ± 5.83 ^c^	18.59 ± 0.35
Linoleic Acid (C18:2 n-6c,9c)	536.76 ± 17.39 ^a^	539.15 ± 15.06 ^a^	538.16 ± 3.10 ^a^	51.65 ± 1.63	230.69 ± 10.21 ^b^	42.68 ± 0.84	373.85 ± 13.67 ^c^	50.81 ± 1.58	315.90 ± 11.17 ^d^	47.87 ± 0.34
α-Linolenic Acid (C18:3 n-3c,6c,9c)	1.21 ± 0.04 ^a^	1.26 ± 0.05 ^a^	1.25 ± 0.10 ^a^	0.12 ± 0.00	*nd*	—	0.73 ± 0.10 ^b^	0.10 ± 0.01	*nd*	—
Arachidic Acid (C20:0)	24.37 ± 2.13 ^a^	24.42 ± 2.71 ^a^	24.97 ± 0.10 ^a^	2.34 ± 0.19	16.32 ± 0.93 ^b^	3.02 ± 0.09	15.60 ± 0.81 ^b^	2.12 ± 0.11	13.56 ± 0.09 ^b^	2.05 ± 0.17
Gondoic Acid (C20:1 n-9c)	22.35 ± 2.41 ^a^	23.46 ± 2.60 ^a^	24.69 ± 0.25 ^a^	2.15 ± 0.26	11.73 ± 1.74 ^b^	2.17 ± 0.29	14.68 ± 1.01 ^c^	2.00 ± 0.10	16.44 ± 0.84 ^c^	2.49 ± 0.06
Eicosadienoic Acid (C20:2 n-6c,11c)	2.13 ± 0.12 ^a^	2.28 ± 0.13 ^a^	2.31 ± 0.22 ^a^	0.20 ± 0.01	0.19 ± 0.03 ^b^	0.04 ± 0.02	1.10 ± 0.12 ^c^	0.15 ± 0.01	0.78 ± 0.09 ^b^	0.12 ± 0.07
Behenic Acid (C22:0)	11.29 ± 0.59 ^a^	11.27 ± 0.26 ^a^	11.12 ± 0.50 ^a^	1.09 ± 0.06	4.67 ± 0.60 ^b^	0.86 ± 0.09	6.76 ± 0.80 ^c^	0.92 ± 0.09	7.49 ± 0.66 ^c^	1.14 ± 0.06
Erucic Acid (C22:1 n-9c)	0.13 ± 0.02 ^a^	0.16 ± 0.05 ^a^	0.19 ± 0.02 ^a^	0.01 ± 0.00	*nd*	—	0.10 ± 0.01 ^b^	0.01 ± 0.01	*nd*	—
Nervonic Acid (C24:1 n-9c)	0.08 ± 0.01 ^a^	0.08 ± 0.01 ^a^	0.09 ± 0.03 ^a^	0.01 ± 0.00	*nd*	—	*nd*	—	*nd*	—
SFA	264.19 ± 7.97 ^a^	260.21 ± 5.85 ^a^	253.55 ± 3.47 ^a^	25.42 ± 0.48	190.37 ± 4.22 ^b^	35.22 ± 4.22	208.43 ± 7.43 ^c^	28.33 ± 0.75	200.84 ± 3.32 ^bc^	30.44 ± 0.47
MUFA	234.84 ± 13.03 ^a^	223.46 ± 8.55 ^a^	215.89 ± 3.18 ^a^	22.60 ± 1.15	119.38 ± 7.54 ^b^	22.08 ± 1.23	151.63 ± 6.99 ^c^	20.61 ± 0.84	142.64 ± 6.46 ^d^	21.62 ± 0.34
PUFA	540.10 ± 17.25 ^a^	542.69 ± 15.16 ^a^	541.72 ± 14.00 ^a^	51.98 ± 1.62	230.82 ± 10.31 ^b^	42.70 ± 0.85	375.67 ± 0.16 ^c^	51.06 ± 1.59	316.41 ± 11.51 ^d^	47.95 ± 0.34
Total	1039.13 ± 17.89 ^a^	1026.35 ± 12.52 ^a^	1011.17 ± 7.98 ^a^		540.57 ± 15.88 ^b^		735.74 ± 15.75 ^c^		659.90 ± 20.96 ^d^	
UFA/SFA	2.93 ± 0.07 ^a^	2.94 ± 0.04 ^a^	2.99 ± 0.09 ^a^		1.84 ± 0.06 ^b^		2.53 ± 0.09 ^c^		2.29 ± 0.05 ^d^	

Values are means of three replicates ± standard deviation. Different lowercase letters (^a^, ^b^, ^c^, and ^d^) indicate statistically significant differences (*p* ≤ 0.05) between all treatment variants and storage times within the same compound and mushroom species, according to Tukey’s HSD test. Abbreviation: *nd*—not detected. SFA—saturated fatty acids; MUFA—monounsaturated fatty acids; PUFA—polyunsaturated fatty acids; UFA/SFA—ratio between unsaturated and saturated fatty acids.

**Table 6:** Average content (mg/100 g d.w.) of free fatty acids in *B. edulis* before the treatment and storage (0 h) and after 96 h of storage.

		*Boletus edulis*		
		0 h	96 h	Relative Change
Lauric Acid (C12:0)	Control	0.23 ± 0.04 ^a^	0.25 ± 0.03 ^a^	—
	Fennel	0.27 ± 0.08 ^ab^	0.38 ± 0.04 ^b^	—
	Spruce	0.30 ± 0.07 ^ab^	0.23 ± 0.03 ^a^	—
Myristic Acid (C14:0)	Control	0.53 ± 0.08 ^a^	2.11 ± 0.15 ^b^	+298%
	Fennel	0.53 ± 0.10 ^a^	1.62 ± 0.19 ^c^	+205%
	Spruce	0.56 ± 0.10 ^a^	0.99 ± 0.22 ^d^	+77%
Pentadecanoic Acid (C15:0)	Control	*nd*	0.58 ± 0.05 ^a^	—
	Fennel	*nd*	0.84 ± 0.10 ^b^	—
	Spruce	*nd*	*nd*	—
Palmitic Acid (C16:0)	Control	6.54 ± 0.29 ^a^	10.17 ± 0.27 ^ab^	—
	Fennel	7.40 ± 0.67 ^ab^	12.47 ± 2.09 ^ab^	—
	Spruce	7.58 ± 0.60 ^ab^	8.63 ± 0.68 ^b^	—
Palmitoleic Acid (C16:1 n-7c)	Control	0.69 ± 0.14 ^a^	2.94 ± 0.08 ^b^	+326%
	Fennel	0.71 ± 0.12 ^a^	0.82 ± 0.09 ^a^	—
	Spruce	0.73 ± 0.05 ^a^	1.27 ± 0.08 ^c^	+74%
Palmitelaidic Acid (C16:1 n-7t)	Control	*nd*	0.55 ± 0.10 ^a^	—
	Fennel	*nd*	0.90 ± 0.07 ^b^	—
	Spruce	*nd*	1.45 ± 0.12 ^c^	—
Margaric Acid (C17:0)	Control	*nd*	6.53 ± 0.44	—
	Fennel	*nd*	*nd*	—
	Spruce	*nd*	*nd*	—
Stearic Acid (C18:0)	Control	7.98 ± 0.60 ^a^	13.47 ± 0.90 ^b^	+69%
	Fennel	8.01 ± 0.36 ^a^	11.33 ± 0.97 ^c^	+41%
	Spruce	7.98 ± 0.22 ^a^	8.62 ± 0.58 ^b^	+8%
Oleic Acid (C18:1 n-9c)	Control	34.28 ± 2.02 ^a^	69.34 ± 2.22 ^b^	+102%
	Fennel	34.02 ± 1.97 ^a^	56.07 ± 2.80 ^c^	+65%
	Spruce	33.33 ± 3.56 ^a^	48.33 ± 0.93 ^c^	+45%
Linoleic Acid (C18:2 n-6c,9c)	Control	75.79 ± 3.60 ^ab^	108.66 ± 4.67 ^c^	+43%
	Fennel	74.73 ± 2.46 ^a^	92.70 ± 5.69 ^d^	+24%
	Spruce	74.82 ± 4.33 ^ab^	88.33 ± 3.45 ^bd^	—
Arachidic Acid (C20:0)	Control	3.41 ± 0.18 ^a^	5.50 ± 0.42 ^b^	+61%
	Fennel	3.43 ± 0.21 ^a^	4.83 ± 0.19 ^bc^	+41%
	Spruce	3.44 ± 0.22 ^a^	4.30 ± 0.15 ^ac^	—
Gondoic Acid (C20:1 n-9c)	Control	1.70 ± 0.13 ^a^	2.38 ± 0.20 ^b^	—
	Fennel	1.74 ± 0.10 ^a^	2.23 ± 0.11 ^b^	—
	Spruce	1.70 ± 0.10 ^a^	2.18 ± 0.07 ^b^	—
Total	Control	131.16 ± 4.73 ^a^	222.48 ± 7.80 ^b^	+69%
	Fennel	130.83 ± 2.56 ^a^	184.20 ± 2.79 ^c^	+41%
	Spruce	130.43 ± 2.86 ^a^	164.33 ± 3.32 ^d^	+26%

Values are means of three replicates ± standard deviation. Different lowercase letters (^a^, ^b^, ^c^, and ^d^) indicate statistically significant differences (*p* ≤ 0.05) between all treatment variants and storage times within the same compound and mushroom species, according to Tukey’s HSD test. Abbreviation: *nd*—not detected.

**Table 7:** Average content (mg/100 g d.w.) of free fatty acids in *I. badia* before the treatment and storage (0 h) and after 96 h of storage.

		*Imleria badia*		
		0 h	96 h	Relative Change
Lauric Acid (C12:0)	Control	0.40 ± 0.06 ^a^	0.46 ± 0.05 ^a^	—
	Fennel	0.40 ± 0.03 ^a^	0.47 ± 0.03 ^a^	—
	Spruce	0.40 ± 0.07 ^a^	0.47 ± 0.05 ^a^	—
Myristic Acid (C14:0)	Control	1.74 ± 0.12 ^a^	1.99 ± 0.08 ^a^	—
	Fennel	1.77 ± 0.14 ^a^	1.95 ± 0.07 ^a^	—
	Spruce	1.72 ± 0.05 ^a^	1.82 ± 0.08 ^a^	—
Pentadecanoic Acid (C15:0)	Control	0.68 ± 0.07 ^a^	2.93 ± 0.07 ^b^	+331%
	Fennel	0.69 ± 0.07 ^a^	1.39 ± 0.11 ^c^	+48%
	Spruce	0.68 ± 0.05 ^a^	3.03 ± 0.15 ^b^	+345%
Palmitic Acid (C16:0)	Control	30.31 ± 3.94 ^a^	42.81 ± 1.38 ^b^	+41%
	Fennel	30.20 ± 0.96 ^a^	37.21 ± 3.03 ^c^	+23%
	Spruce	30.35 ± 1.11 ^a^	49.38 ± 3.83 ^d^	+63%
Palmitoleic Acid (C16:1 n-7c)	Control	1.05 ± 0.07 ^a^	2.47 ± 0.18 ^b^	+135%
	Fennel	1.06 ± 0.14 ^a^	2.41 ± 0.17 ^b^	+127%
	Spruce	1.13 ± 0.06 ^a^	4.13 ± 0.15 ^c^	+265%
Palmitelaidic Acid (C16:1 n-7t)	Control	0.27 ± 0.07 ^a^	0.37 ± 0.05 ^b^	+37%
	Fennel	0.23 ± 0.06 ^a^	0.28 ± 0.04 ^b^	+22%
	Spruce	0.26 ± 0.04 ^a^	0.62 ± 0.07 ^c^	+138%
Margaric Acid (C17:0)	Control	*nd*	0.32 ± 0.04 ^ab^	—
	Fennel	*nd*	0.21 ± 0.05 ^b^	—
	Spruce	*nd*	0.53 ± 0.07 ^a^	—
Stearic Acid (C18:0)	Control	10.51 ± 0.69 ^a^	28.93 ± 2.30 ^b^	+175%
	Fennel	10.57 ± 0.65 ^a^	17.06 ± 2.51 ^c^	+59%
	Spruce	10.73 ± 0.82 ^a^	27.42 ± 3.19 ^b^	+156%
Oleic Acid (C18:1 n-9c)	Control	50.41 ± 3.01 ^a^	98.78 ± 6.32 ^b^	+96%
	Fennel	50.49 ± 3.20 ^a^	78.48 ± 6.19 ^c^	+55%
	Spruce	50.60 ± 3.19 ^a^	98.44 ± 6.98 ^b^	+94%
Linoleic Acid (C18:2 n-6c,9c)	Control	85.07 ± 4.54 ^a^	209.40 ± 9.51 ^b^	+146%
	Fennel	87.08 ± 3.40 ^a^	117.70 ± 8.51 ^c^	+35%
	Spruce	84.43 ± 3.33 ^a^	187.00 ± 4.42 ^d^	+121%
Total	Control	181.53 ± 5.77 ^a^	388.45 ± 1.93 ^b^	+114%
	Fennel	182.18 ± 3.92 ^a^	257.15 ± 7.72 ^c^	+41%
	Spruce	180.31 ± 6.21 ^a^	372.84 ± 4.25 ^d^	+107%

Values are means of three replicates ± standard deviation. Different lowercase letters (^a^, ^b^, ^c^, and ^d^) indicate statistically significant differences (*p* ≤ 0.05) between all treatment variants and storage times within the same compound and mushroom species, according to Tukey’s HSD test. Abbreviation: *nd*—not detected.

**Table 8:** Average content (mg/100 g d.w.) of free fatty acids in *A. bisporus* before the treatment and storage (0 h) and after 96 h of storage.

		*Agaricus bisporus*		
		0 h	96 h	Relative Change
Myristic Acid (C14:0)	Control	3.10 ± 0.12 ^a^	3.70 ± 0.13 ^a^	—
	Fennel	3.02 ± 0.12 ^a^	3.69 ± 0.17 ^a^	—
	Spruce	3.04 ± 0.06 ^a^	3.15 ± 0.06 ^a^	—
Pentadecanoic Acid (C15:0)	Control	9.15 ± 0.14 ^a^	11.35 ± 0.38 ^b^	+24%
	Fennel	9.04 ± 0.07 ^a^	9.70 ± 0.29 ^c^	+7%
	Spruce	9.05 ± 0.08 ^a^	9.80 ± 0.20 ^b^	+8%
Palmitic Acid (C16:0)	Control	23.89 ± 2.18 ^a^	32.01 ± 2.43 ^b^	+34%
	Fennel	22.46 ± 1.01 ^a^	25.33 ± 1.07 ^c^	+13%
	Spruce	22.96 ± 1.47 ^a^	24.77 ± 1.94 ^d^	+8%
Palmitoleic Acid (C16:1 n-7c)	Control	0.72 ± 0.07 ^ab^	0.88 ± 0.03 ^a^	—
	Fennel	0.73 ± 0.04 ^ab^	0.74 ± 0.03 ^ab^	—
	Spruce	0.75 ± 0.06 ^ab^	0.68 ± 0.02 ^b^	—
Margaric Acid (C17:0)	Control	*nd*	1.47 ± 0.14a ^b^	—
	Fennel	*nd*	0.59 ± 0.14 ^b^	—
	Spruce	*nd*	0.52 ± 0.15 ^a^	—
Stearic Acid (C18:0)	Control	17.53 ± 0.30 ^a^	24.54 ± 1.88 ^b^	+40%
	Fennel	17.56 ± 0.39 ^a^	19.23 ± 0.31 ^c^	+10%
	Spruce	17.18 ± 0.18 ^a^	18.45 ± 0.51 ^b^	+7%
Oleic Acid (C18:1 n-9c)	Control	33.98 ± 2.21 ^a^	54.51 ± 3.58 ^b^	+61%
	Fennel	32.44 ± 1.29 ^a^	40.63 ± 1.58 ^c^	+25%
	Spruce	32.79 ± 1.13 ^a^	40.80 ± 2.22 ^c^	+24%
Linoleic Acid (C18:2 n-6c,9c)	Control	85.07 ± 4.54 ^a^	179.40 ± 9.51 ^b^	+111%
	Fennel	86.08 ± 3.40 ^a^	117.70 ± 8.51 ^c^	+37%
	Spruce	84.43 ± 3.33 ^a^	143.66 ± 3.16 ^d^	+70%
Arachidic Acid (C20:0)	Control	6.16 ± 0.45 ^a^	10.14 ± 0.79 ^b^	+65%
	Fennel	5.86 ± 0.60 ^a^	8.68 ± 0.68 ^bc^	+48%
	Spruce	6.05 ± 0.48 ^a^	7.66 ± 0.46 ^c^	+27%
Gondoic Acid (C20:1 n-9c)	Control	2.49 ± 0.21 ^ab^	10.56 ± 0.69 ^c^	+324%
	Fennel	2.26 ± 0.16 ^a^	2.79 ± 0.13 ^b^	+23%
	Spruce	2.22 ± 0.13 ^a^	3.47 ± 0.32 ^d^	+56%
Behenic Acid (C22:0)	Control	1.49 ± 0.26 ^a^	2.54 ± 0.24 ^c^	+70%
	Fennel	1.50 ± 0.36 ^a^	2.31 ± 0.17 ^bc^	+54%
	Spruce	1.54 ± 0.30 ^ab^	2.19 ± 0.17 ^abc^	+42%
Total	Control	180.13 ± 5.86 ^a^	331.09 ± 7.99 ^b^	+84%
	Fennel	180.94 ± 6.20 ^a^	231.37 ± 7.25 ^c^	+28%
	Spruce	180.01 ± 3.23 ^a^	255.17 ± 1.66 ^d^	+42%

Values are means of three replicates ± standard deviation. Different lowercase letters (^a^, ^b^, ^c^, and ^d^) indicate statistically significant differences (*p* ≤ 0.05) between all treatment variants and storage times within the same compound and mushroom species, according to Tukey’s HSD test. Abbreviation: *nd*—not detected.

**Table 9:** The average content of tocopherols (mg/100 g d.w) in *B. edulis*, *I. badia* and *A. bisporus* before the treatment and storage (0 h) and after 96 h of storage.

		*Boletus edulis*		*Imleria badia*		*Agaricus bisporus*	
		0 h	96 h	0 h	96 h	0 h	96 h
α-tocopherol	Control	0.262 ± 0.013 ^a^	0.016 ± 0.002 ^b^	0.553 ± 0.014 ^a^	0.392 ± 0.015 ^b^	0.148 ± 0.005 ^a^	0.019 ± 0.002 ^b^
	Fennel	0.261 ± 0.021 ^a^	0.075 ± 0.006 ^c^	0.542 ± 0.021 ^a^	0.066 ± 0.007 ^c^	0.146 ± 0.007 ^a^	0.007 ± 0.003 ^b^
	Spruce	0.267 ± 0.022 ^a^	0.182 ± 0.008 ^d^	0.544 ± 0.011 ^a^	0.135 ± 0.007 ^d^	0.149 ± 0.005 ^a^	0.015 ± 0.003 ^b^
β-tocopherol	Control	0.347 ± 0.010 ^a^	0.019 ± 0.001 ^b^	0.716 ± 0.005 ^a^	0.056 ± 0.003 ^b^	0.230 ± 0.011 ^a^	0.027 ± 0.002 ^b^
	Fennel	0.338 ± 0.009 ^a^	0.051 ± 0.006 ^c^	0.718 ± 0.014 ^a^	0.053 ± 0.008 ^b^	0.219 ± 0.002 ^a^	0.060 ± 0.002 ^c^
	Spruce	0.336 ± 0.013 ^a^	0.010 ± 0.003 ^b^	0.714 ± 0.005 ^a^	0.043 ± 0.004 ^b^	0.221 ± 0.006 ^a^	0.014 ± 0.003 ^b^
γ-tocopherol	Control	0.738 ± 0.003 ^a^	0.260 ± 0.033 ^b^	1.493 ± 0.010 ^a^	1.035 ± 0.015 ^b^	0.231 ± 0.002 ^a^	0.105 ± 0.003 ^b^
	Fennel	0.744 ± 0.006 ^a^	0.473 ± 0.011 ^c^	1.492 ± 0.009 ^a^	1.481 ± 0.024 ^a^	0.238 ± 0.014 ^a^	0.120 ± 0.002 ^b^
	Spruce	0.764 ± 0.014 ^a^	0.268 ± 0.024 ^b^	1.493 ± 0.009 ^a^	0.978 ± 0.028 ^b^	0.235 ± 0.007 ^a^	0.137 ± 0.004 ^b^
δ-tocopherol	Control	0.475 ± 0.016 ^ab^	0.158 ± 0.004 ^c^	1.027 ± 0.020 ^a^	0.123 ± 0.007 ^b^	0.107 ± 0.004 ^a^	0.089 ± 0.003 ^a^
	Fennel	0.489 ± 0.010 ^a^	0.154 ± 0.007 ^c^	1.035 ± 0.020 ^a^	0.117 ± 0.006 ^b^	0.106 ± 0.002 ^a^	0.103 ± 0.001 ^a^
	Spruce	0.457 ± 0.017 ^b^	0.289 ± 0.004 ^d^	1.035 ± 0.025 ^a^	0.093 ± 0.003 ^c^	0.105 ± 0.006 ^a^	0.094 ± 0.005 ^a^
Sum of tocopherols	Control	1.822 ± 0.027 ^a^	0.453 ± 0.037 ^b^	3.788 ± 0.022 ^a^	1.605 ± 0.011 ^b^	0.716 ± 0.013 ^a^	0.241 ± 0.007 ^b^
	Fennel	1.832 ± 0.023 ^a^	0.753 ± 0.022 ^c^	3.788 ± 0.035 ^a^	1.717 ± 0.031 ^c^	0.710 ± 0.017 ^a^	0.290 ± 0.008 ^c^
	Spruce	1.824 ± 0.008 ^a^	0.750 ± 0.020 ^c^	3.786 ± 0.019 ^a^	1.248 ± 0.038 ^d^	0.711 ± 0.007 ^a^	0.260 ± 0.004 ^bc^

Values are means of three replicates ± standard deviation. Different lowercase letters (^a^, ^b^, ^c^, and ^d^) indicate statistically significant differences (*p* ≤ 0.05) between all treatment variants and storage times within the same compound and mushroom species, according to Tukey’s HSD test.

**Table 10:** The average content of vitamins B_1_, B_2_, and B_6_ (mg/100 g d.w) in *B. edulis*, *I. badia* and *A. bisporus* before the treatment and storage (0 h) and after 96 h of storage.

		*Boletus edulis*		*Imleria badia*		*Agaricus bisporus*	
		0 h	96 h	0 h	96 h	0 h	96 h
Vitamin B_1_	Control	6.60 ± 0.372 ^a^	4.50 ± 2.7 ^b^	5.94 ± 0.27 ^a^	2.86 ± 0.16 ^b^	3.66 ± 0.15 ^a^	2.71 ± 0.12 ^b^
	Fennel	6.63 ± 0.382 ^a^	6.27 ± 1.8 ^ac^	5.99 ± 0.32 ^a^	4.78 ± 0.23 ^c^	3.63 ± 0.13 ^a^	3.30 ± 0.14 ^ac^
	Spruce	6.53 ± 0.208 ^a^	5.56 ± 3.26 ^c^	5.89 ± 0.35 ^a^	4.85 ± 0.16 ^c^	3.70 ± 0.13 ^a^	2.99 ± 0.12 ^bc^
Vitamin B_2_	Control	30.09 ± 2.084 ^a^	22.80 ± 19.65 ^b^	32.92 ± 2.33 ^a^	23.69 ± 1.83 ^b^	21.90 ± 1.29 ^a^	18.82 ± 1.33 ^ab^
	Fennel	30.11 ± 2.032 ^a^	20.21 ± 11.29 ^b^	33.40 ± 1.90 ^a^	20.04 ± 1.71 ^b^	22.08 ± 1.11 ^a^	18.06 ± 1.51 ^b^
	Spruce	29.75 ± 3.595 ^a^	23.83 ± 13.45 ^b^	32.30 ± 2.05 ^a^	30.41 ± 1.57 ^a^	22.11 ± 1.00 ^a^	19.15 ± 1.04 ^ab^
Vitamin B_6_	Control	0.117 ± 0.007 ^a^	0.067 ± 0.001 ^b^	0.166 ± 0.004 ^a^	0.138 ± 0.002 ^b^	0.074 ± 0.005 ^a^	0.035 ± 0.003 ^b^
	Fennel	0.113 ± 0.004 ^a^	0.094 ± 0.002 ^c^	0.166 ± 0.004 ^a^	0.113 ± 0.003 ^c^	0.073 ± 0.005 ^a^	0.051 ± 0.003 ^c^
	Spruce	0.116 ± 0.003 ^a^	0.066 ± 0.001 ^b^	0.166 ± 0.003 ^a^	0.115 ± 0.001 ^c^	0.073 ± 0.004 ^a^	0.041 ± 0.002 ^b^

Values are means of three replicates ± standard deviation. Different lowercase letters indicate statistically significant differences (*p* ≤ 0.05) between all treatment variants and storage times within the same compound and mushroom species, according to Tukey’s HSD test.

## Data Availability

The original contributions presented in the study are included in the article; further inquiries can be directed to the corresponding author.

## Supplementary Materials

The following supporting information can be downloaded at https://www.mdpi.com/article/10.3390/ijms26093939/s1.

## References

[1] Tan Y., Zeng N.K., Xu B. (2022). Chemical Profiles and Health-Promoting Effects of Porcini Mushroom (Boletus Edulis): A Narrative Review. Food Chem..

[2] Phillips J.M., Ooi S.L., Pak S.C. (2022). Health-Promoting Properties of Medicinal Mushrooms and Their Bioactive Compounds for the COVID-19 Era—An Appraisal: Do the Pro-Health Claims Measure Up?. Molecules.

[3] Mahajan P.V., Oliveira F.A.R., Macedo I. (2008). Effect of Temperature and Humidity on the Transpiration Rate of the Whole Mushrooms. J. Food Eng..

[4] Perumal A.B., Sellamuthu P.S., Nambiar R.B., Sadiku E.R. (2017). Effects of Essential Oil Vapour Treatment on the Postharvest Disease Control and Different Defence Responses in Two Mango (*Mangifera indica* L.) Cultivars. Food Bioproc Technol..

[5] Wang C.Y., Wang S.Y., Yin J.J., Parry J., Yu L.L. (2007). Enhancing Antioxidant, Antiproliferation, and Free Radical Scavenging Activities in Strawberries with Essential Oils. J. Agric. Food Chem..

[6] Spisni E., Petrocelli G., Imbesi V., Spigarelli R., Azzinnari D., Sarti M.D., Campieri M., Valerii M.C. (2020). Antioxidant, Anti-Inflammatory, and Microbial-Modulating Activities of Essential Oils: Implications in Colonic Pathophysiology. Int. J. Mol. Sci..

[7] Shahat A.A., Ibrahim A.Y., Hendawy S.F., Omer E.A., Hammouda F.M., Abdel-Rahman F.H., Saleh M.A. (2011). Chemical Composition, Antimicrobial and Antioxidant Activities of Essential Oils from Organically Cultivated Fennel Cultivars. Molecules.

[8] Schoss K., Kočevar Glavač N., Kreft S. (2023). Volatile Compounds in Norway Spruce (*Picea abies*) Significantly Vary with Season. Plants.

[9] Gao M., Feng L., Jiang T. (2014). Browning Inhibition and Quality Preservation of Button Mushroom (*Agaricus bisporus*) by Essential Oils Fumigation Treatment. Food Chem..

[10] Aly A.A., Mohammed M.K., Maraei R.W., Abdalla A.E., Abouel-Yazeed A.M. (2023). Improving the Nutritional Quality and Bio-Ingredients of Stored White Mushrooms Using Gamma Irradiation and Essential Oils Fumigation. Radiochim. Acta.

[11] Jiang T., Luo Z., Ying T. (2015). Fumigation with Essential Oils Improves Sensory Quality and Enhanced Antioxidant Ability of Shiitake Mushroom (*Lentinus edodes*). Food Chem..

[12] Wang Y., Wang Y., Wang K., Cheng M., Zhao P., Lu J., Xi X., Wang X., Han X., Wang J. (2024). Evaluation of the Postharvest Quality of Agaricus Bisporus Packed Using PVA/SG-Based Active Packaging Film Containing Tea Tree Essential Oil. J. Food Meas. Charact..

[13] Nasiri M., Barzegar M., Sahari M.A., Niakousari M. (2018). Application of Tragacanth Gum Impregnated with Satureja Khuzistanica Essential Oil as a Natural Coating for Enhancement of Postharvest Quality and Shelf Life of Button Mushroom (*Agaricus bisporus*). Int. J. Biol. Macromol..

[14] Karimirad R., Behnamian M., Dezhsetan S., Sonnenberg A. (2018). Chitosan Nanoparticles-Loaded Citrus Aurantium Essential Oil: A Novel Delivery System for Preserving the Postharvest Quality of Agaricus Bisporus. J. Sci. Food Agric..

[15] Garg N., Singh V.K. (2013). Carbohydrate Metabolism During Fruit Spoilage. Biotechnology in Horticulture: Methods and Applications.

[16] Yu L., Liu H., Shao X., Yu F., Wei Y., Ni Z., Xu F., Wang H. (2016). Effects of Hot Air and Methyl Jasmonate Treatment on the Metabolism of Soluble Sugars in Peach Fruit during Cold Storage. Postharvest Biol. Technol..

[17] Fernandez O., Béthencourt L., Quero A., Sangwan R.S., Clément Christophe C. (2010). Trehalose and Plant Stress Responses: Friend or Foe?. Trends Plant Sci..

[18] Shen B., Hohmann S., Jensen R.G., Bohnert H.J. (1999). Roles of Sugar Alcohols in Osmotic Stress Adaptation. Replacement of Glycerol by Mannitol and Sorbitol in Yeast 1. Plant Physiol..

[19] Chen Y.-Z., Zhang B.W., Yang J., Zou C.S., Li T., Zhang G.C., Chen G.S. (2021). Detoxification, Antioxidant, and Digestive Enzyme Activities and Gene Expression Analysis of *Lymantria dispar* Larvae under Carvacrol. J. Asia Pac. Entomol..

[20] Jiang L.L., Wang J.B., Wang W.H., Lei B., Feng J.T., Wu H., Ma Z.Q. (2023). Effects of Three Essential Oil Fumigation Treatments on the Postharvest Control of Botrytis Cinerea and Their Efficacy as Preservatives of Cherry Tomatoes. Plant Dis..

[21] Gałgowska M., Pietrzak-Fiećko R. (2022). Evaluation of the Nutritional and Health Values of Selected Polish Mushrooms Considering Fatty Acid Profiles and Lipid Indices. Molecules.

[22] Kapoor B., Kapoor D., Gautam S., Singh R., Bhardwaj S. (2021). Dietary Polyunsaturated Fatty Acids (PUFAs): Uses and Potential Health Benefits. Curr. Nutr. Rep..

[23] Maki K.C., Dicklin M.R., Kirkpatrick C.F. (2021). Saturated Fats and Cardiovascular Health: Current Evidence and Controversies. J. Clin. Lipidol..

[24] Legrand P., Rioux V. (2010). The Complex and Important Cellular and Metabolic Functions of Saturated Fatty Acids. Lipids.

[25] Chaula D., Jacobsen C., Laswai H.S., Chove B.E., Dalsgaard A., Mdegela R., Hyldig G. (2023). Changes in Fatty Acids during Storage of Artisanal-Processed Freshwater Sardines (*Rastrineobola argentea*). Food Sci. Nutr..

[26] Baysal T., Demirdöven A. (2007). Lipoxygenase in Fruits and Vegetables: A Review. Enzym. Microb. Technol..

[27] Farhoosh R., Einafshar S., Sharayei P. (2009). The Effect of Commercial Refining Steps on the Rancidity Measures of Soybean and Canola Oils. Food Chem..

[28] Emebu S., Osaikhuiwuomwan O., Mankonen A., Udoye C., Okieimen C., Janáčová D. (2022). Influence of Moisture Content, Temperature, and Time on Free Fatty Acid in Stored Crude Palm Oil. Sci. Rep..

[29] Marçal S., Sousa A.S., Taofiq O., Antunes F., Morais A.M.M.B., Freitas A.C., Barros L., Ferreira I.C.F.R., Pintado M. (2021). Impact of Postharvest Preservation Methods on Nutritional Value and Bioactive Properties of Mushrooms. Trends Food Sci. Technol..

[30] Cardoso R.V.C., Fernandes Â., Barreira J.C.M., Verde S.C., Antonio A.L., Gonzaléz-Paramás A.M., Barros L., Ferreira I.C.F.R. (2019). Effectiveness of Gamma and Electron Beam Irradiation as Preserving Technologies of Fresh Agaricus Bisporus Portobello: A Comparative Study. Food Chem..

[31] Heleno S.A., Ferreira R.C., Antonio A.L., Queiroz M.J.R.P., Barros L., Ferreira I.C.F.R. (2015). Nutritional Value, Bioactive Compounds and Antioxidant Properties of Three Edible Mushrooms from Poland. Food Biosci..

[32] Wei A., Shibamoto T. (2010). Antioxidant/Lipoxygenase Inhibitory Activities and Chemical Compositions of Selected Essential Oils. J. Agric. Food Chem..

[33] Mäkinen M. (2002). Lipid Hydroperoxides: Effects of Tocopherols and Ascorbic Acid on Their Formation and Decomposition. Ph.D. Thesis.

[34] Ju J., Picinich S.C., Yang Z., Zhao Y., Suh N., Kong A.N., Yang C.S. (2009). Cancer-Preventive Activities of Tocopherols and Tocotrienols. Carcinogenesis.

[35] Lisa S.A., Obaid M., Khan S., Chowdhury K. (2015). Tocopherol Content of Vegetable Oils/Fats and Their Oxidative Deterioration During Storage AND. World J. Pharm. Pharm. Sci..

[36] Šunić L., Ilić Z.S., Stanojević L., Milenković L., Stanojević J., Kovač R., Milenković A., Cvetković D. (2023). Comparison of the Essential Oil Content, Constituents and Antioxidant Activity from Different Plant Parts during Development Stages of Wild Fennel (*Foeniculum vulgare* Mill.). Horticulturae.

[37] Stevanovic F. (2017). &; Francezon, N.; Stevanovic, T. Chemical Composition of Essential Oil and Hydrosol from Picea Mariana Bark Residue. Bioresources.

[38] Radulescu V., Chifiriuc M., Oprea E. (2011). Chemical Composition and Antimicrobial Activity of Essential Oil from Shoots Spruce (*Picea abies* L). Rev. Chim.

[39] Munné-Bosch S., Alegre L. (2002). The Function of Tocopherols and Tocotrienols in Plants. CRC Crit. Rev. Plant Sci..

[40] Rangsinth P., Sharika R., Pattarachotanant N., Duangjan C., Wongwan C., Sillapachaiyaporn C., Nilkhet S., Wongsirojkul N., Prasansuklab A., Tencomnao T. (2023). Potential Beneficial Effects and Pharmacological Properties of Ergosterol, a Common Bioactive Compound in Edible Mushrooms. Foods.

[41] Jiang Q., Zhang M., Mujumdar A.S. (2020). UV Induced Conversion during Drying of Ergosterol to Vitamin D in Various Mushrooms: Effect of Different Drying Conditions. Trends Food Sci. Technol..

[42] Laird E., Ward M., McSorley E., Strain J.J., Wallace J. (2010). Vitamin D and Bone Health; Potential Mechanisms. Nutrients.

[43] Wu Z., Liu D., Deng F. (2022). The Role of Vitamin D in Immune System and Inflammatory Bowel Disease. J. Inflamm. Res..

[44] Yap P.S.X., Yusoff K., Lim S.H.E., Chong C.M., Lai K.S. (2021). Membrane Disruption Properties of Essential Oils-a Double-Edged Sword?. Processes.

[45] Ferreira V.R.F., Militani I.A., de Almeida K.J., Lunguinho A.d.S., Saczk A.A., Ionta M., da Silva G.Á.F., Felix F.S., Nelson D.L., Cardoso M. (2023). das G. Antioxidant and Cytotoxic Activity of Essential Oils and Their Principal Components: Spectrophotometric, Voltammetric, and Theoretical Investigation of the Chelating Effect of Eugenol and Carvacrol. ACS Food Sci. Technol..

[46] Guo Y., Chen X., Gong P., Wang R., Han A., Deng Z., Qi Z., Long H., Wang J., Yao W. (2023). Advances in the Role and Mechanisms of Essential Oils and Plant Extracts as Natural Preservatives to Extend the Postharvest Shelf Life of Edible Mushrooms. Foods.

[47] Said H.M. (2011). Intestinal Absorption of Water-Soluble Vitamins in Health and Disease. Biochem. J..

[48] Giannakourou M.C., Taoukis P.S. (2021). Effect of Alternative Preservation Steps and Storage on Vitamin c Stability in Fruit and Vegetable Products: Critical Review and Kinetic Modelling Approaches. Foods.

[49] Bernaś E., Jaworska G. (2016). Vitamins Profile as an Indicator of the Quality of Frozen Agaricus Bisporus Mushrooms. J. Food Compos. Anal..

[50] Geransayeh M., Sepahvand S., Abdossi V., Nezhad R.A. (2015). Effect of Thymol Treatment on Decay, Postharvest Life and Quality of Strawberry (*Fragaria ananassa*) Fruit Cv. “Gaviota”. Int. J. Agron. Agric. Res..

[51] Santoro K., Maghenzani M., Chiabrando V., Bosio P., Gullino M.L., Spadaro D., Giacalone G. (2018). Thyme and Savory Essential Oil Vapor Treatments Control Brown Rot and Improve the Storage Quality of Peaches and Nectarines, but Could Favor Gray Mold. Foods.

[52] Namiota M., Bonikowski R. (2021). The Current State of Knowledge about Essential Oil Fumigation for Quality of Crops during Postharvest. Int. J. Mol. Sci..

[53] Freire R.S., Morais S.M., Catunda F.E.A., Pinheiro D.C.S.N. (2005). Synthesis and Antioxidant, Anti-Inflammatory and Gastroprotector Activities of Anethole and Related Compounds. Bioorg Med. Chem..

[54] Ahmed Salim E.R. (2017). Salim-Eisa Method for Modification of Evaporation Test (British Pharmacopeia) by Sudanese Essential Oils. J. Appl. Biotechnol. Bioeng..

[55] Aninowski M., Leszczyńska J., Bonikowski R., Ponder A., Ewelina H., Małgorzata G. (2024). Kamil Szymczak Suitability of Selected Apple Varieties for People with Allergies and Diabetes. Nutrients.

[56] Piatek P., Lewkowicz N., Michlewska S., Wieczorek M., Bonikowski R., Parchem K., Lewkowicz P., Namiecinska M. (2022). Natural Fish Oil Improves the Differentiation and Maturation of Oligodendrocyte Precursor Cells to Oligodendrocytes in Vitro after Interaction with the Blood–Brain Barrier. Front. Immunol..

[57] Cyran M.R., Dynkowska W.M., Ceglińska A., Bonikowski R. (2021). Improving Rye Bread Antioxidant Capacity by Bread-Making Methodology: Contribution of Phosphate-Buffered Saline- and Methanol-Soluble Phenolic Phytochemicals with Different Molecular Profiles. J. Cereal Sci..

[58] Sasaki K., Hatate H., Tanaka R. (2020). Determination of 13 Vitamin B and the Related Compounds Using HPLC with UV Detection and Application to Food Supplements. Chromatographia.

