# Supplementary material for: Essential Oil Fumigation Modulates Nutrient Content in Selected Mushrooms During Postharvest Storage
Source: Int J Mol Sci. 2025 Apr 22;26(9):3939. doi: 10.3390/ijms26093939 (PMC12071996; doi:10.3390/ijms26093939)

Figure S1. Bar-plot presenting the Z-scored distribution of free sugars within samples. Be, Ib and Ab stands for respectively: B. edulis, I. badia and A. bisporus. C, F and S stands for, respectively: control samples, fennel essential oil-fumigated samples and spruce essential oil-fumigated samples.

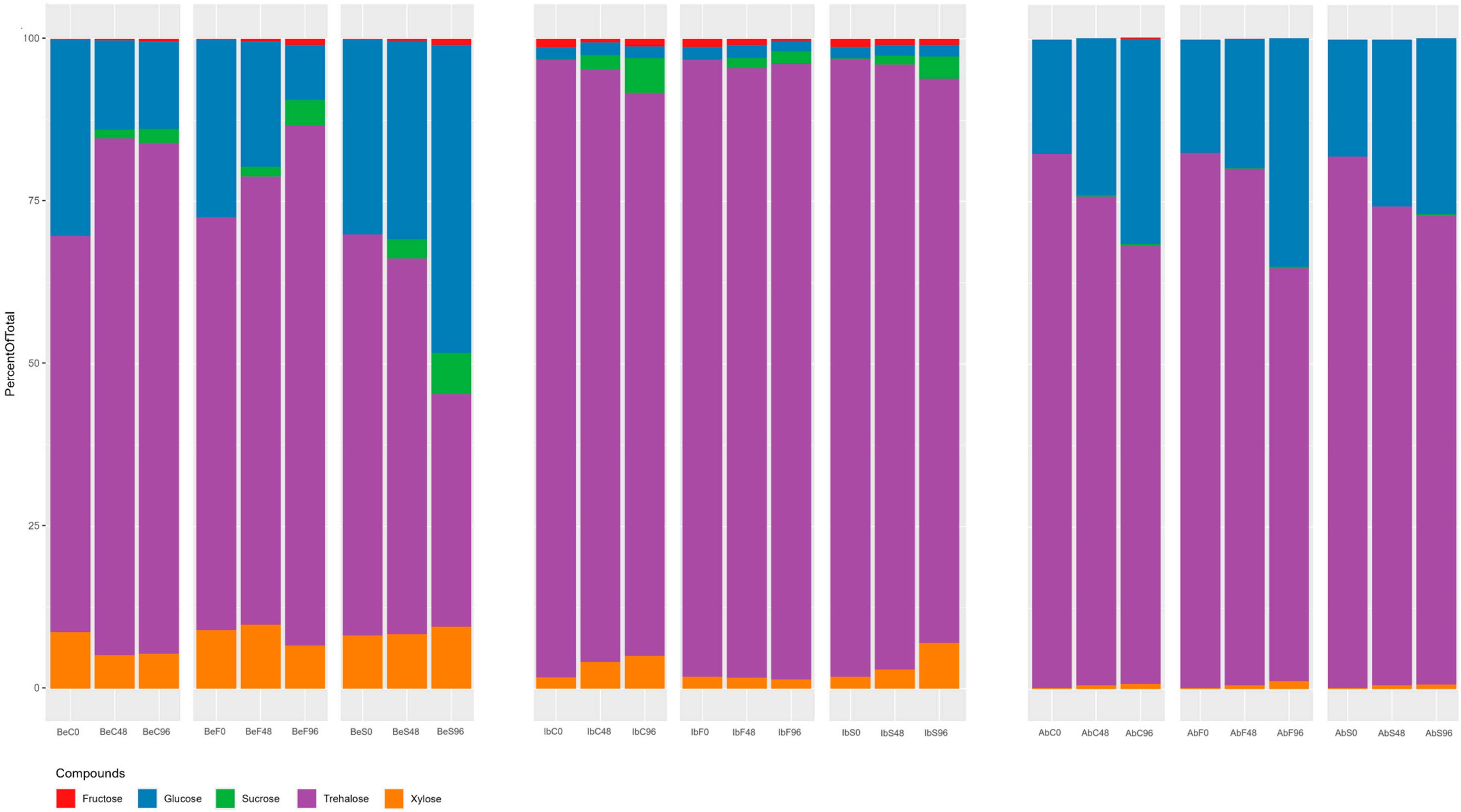

Supplement: Supplementary file 1 [file ijms-26-03939-s001.zip › Figure S1. Bar-plot presenting the Z-scored distribution of free sugars within samples.pdf]
