# Supplementary material for: Essential Oil Fumigation Modulates Nutrient Content in Selected Mushrooms During Postharvest Storage
Source: Int J Mol Sci. 2025 Apr 22;26(9):3939. doi: 10.3390/ijms26093939 (PMC12071996; doi:10.3390/ijms26093939)
Supplement: Supplementary file 1 [file ijms-26-03939-s001.zip › Tables S1-S6. ANOVA type III results.pdf]

Table S1. Statistical significance (*p*-values) from Type III ANOVA *F*-tests for main effects and their interactions on free sugars based on three-factor linear model (Variant × Time × Fungus) and *t*-tests for selected Variant:Time interaction coefficients.

	Glucose	Fructose	Xylose	Trehalose	Sucrose	Total sugars
Time	***	***	***	***	***	***
Variant						
Variant:Time	***	***	**	***	**	***
VariantFennel:Time96		***		***	*	***
VariantSpruce:Time96	*	***				
Fungus	***	***	***	***	***	***
Fungus:Time	***	***	***	***	***	***

Asterisks indicate significant impact of effects and interaction, *0.01 ≤ p<0.05; **0.001 ≤ p<0.01; ***p < 0.001.

Table S2. Statistical significance (*p*-values) from Type III ANOVA *F*-tests for main effects and their interactions on free sugar alcohols based on three-factor linear model (Variant × Time × Fungus) and *t*-tests for selected Variant:Time interaction coefficients.

	Sorbitol	Mannitol	Arabitol	Erythritol	Total sugar alcohols
Time	***	***	***	***	***
Variant					
Variant:Time	***	***	**		***
VariantFennel:Time96	*	**	**		
VariantSpruce:Time96	***		***	*	***
Fungus	***	***	***	***	***
Fungus:Time	***	***	***	***	***

Asterisks indicate significant impact of effects and interaction, *0.01 ≤ p<0.05; **0.001 ≤ p<0.01; ***p < 0.001

Table S3. Statistical significance (*p*-values) from Type III ANOVA *F*-tests for main effects and their interactions on free fatty acids based on three-factor linear model (Variant × Time × Fungus) and *t*-tests for selected Variant:Time interaction coefficients

	C14:0	C15:0	C16:0	C16:1c	C16:1t	C17:0	C18:0	C18:1	C18:2	C20:0	C20:1	C22:0	Total
Time	**	***	***	*	***	***	***	***	***	***	***	***	***
Variant													
Variant:Time		***	*	*	***	***	***	***	***	**	***		***
VariantFennel:Time96		***			***	***	**	***	***		***		***
VariantSpruce:Time96		***	*	*	***	***	**	***	***	**	***		***
Fungus	***	***	***	***	***		***	***	**	***	***	—	***
Fungus:Time	***	***		***	***	***	***	***	***	**	***	—	***

Asterisks indicate significant impact of effects and interaction, *0.01≤p<0.05; **0.001≤p<0.01; ***p<0.001

Table S4. Statistical significance (*p*-values) from Type III ANOVA *F*-tests for main effects and their interactions on fatty acids based on three-factor linear model (Variant × Time × Fungus) and *t*-tests for selected Variant:Time interaction coefficients

	Time	Variant	Variant:Time	VariantFennel:Time96	VariantSpruce:Time96	Fungus	Fungus:Time
C12:0	***		*		**		***
C14:0	***					***	***
C15:0	***					***	**
C16:0	***		***	**	**	***	***
C16:1	***		***	**	***	***	**
C16:2	***		***		***	—	—
C17:0	***		**		**	***	***
C17:1	***		***			—	—
C18:0	***					***	.
C18:1	***	*	***	***	***	***	***
C18:2	***		***	***	***	***	***
C18:3	***		***	***		***	***
C20:0	***					*	**
C20:1	***		*	*	**	***	***
C20:2	***		***	***		***	***
C21:0	***		***	***	***	**	**
C22:0	***		***	**	***	***	**
C22:1	***		**		.	*	
C22:2	***		***	***	***	—	—
C24:1	***					***	***
SFA	***		*	*	**	***	*
MUFA	***		***	***	***	***	***
PUFA	***		***	***	***	***	***
Total	***		***	***	***	***	***
UFA/SFA	***		***	***	***	***	***

Asterisks indicate significant impact of effects and interaction, *0.01≤p<0.05; **0.001≤p<0.01; ***p<0.001.

Table S5. Statistical significance (*p*-values) from Type III ANOVA *F*-tests for main effects and their interactions on tocopherols, vitamin D_2 and ergosterol based on three-factor linear model (Variant × Time × Fungus) and *t*-tests for selected Variant:Time interaction coefficients.

	α-tocopherol	β-tocopherol	γ-tocopherol	δ-tocopherol	Total tocopherols	Ergosterol	Vitamin D_2
Time	***	***	***	*	***	***	***
Variant							
Variant:Time		***			***	***	***
VariantFennel:Time96		***			**	***	***
VariantSpruce:Time96						***	***
Fungus	***	***	***	***	***	***	***
Fungus:Time	***	***	***	***	***	***	***

Asterisks indicate significant impact of effects and interaction, *0.01≤p<0.05; **0.001≤p<0.01; ***p < 0.001.

Table S6. Statistical significance (*p*-values) from Type III ANOVA *F*-tests for main effects and their interactions on water-soluble vitamins based on three-factor linear model (Variant × Time × Fungus) and *t*-tests for selected Variant:Time interaction coefficients.

	Vit. B_1	Vit. B_2	Vit. B_6	Vit. C
Time	***	*	***	***
Variant				
Variant:Time	*		***	***
VariantFennel:Time96	**		***	***
VariantSpruce:Time96			*	***
Fungus	***	***	***	***
Fungus:Time	***		***	***

Asterisks indicate significant impact of effects and interaction, *$0.01 \le p < 0.05$; **$0.001 \le p < 0.01$; ***$p < 0.001$.
