# Supplementary material for: Essential Oil Fumigation Modulates Nutrient Content in Selected Mushrooms During Postharvest Storage
Source: Int J Mol Sci. 2025 Apr 22;26(9):3939. doi: 10.3390/ijms26093939 (PMC12071996; doi:10.3390/ijms26093939)
Supplement: Supplementary file 1 [file ijms-26-03939-s001.zip › Tables S7-S8. Essential oils composition Figures S3-S4 Essential oils composition chromatograms.pdf]

Analysis of the composition of the essential oils

Method:

The analysis of both essential oils was conducted using gas chromatography in Thermo Ultra GC Trace equipped with a flame ionization detector and Thermo DSQ II mass spectrometer (split flow) (Thermo Fisher Scientific, Waltham, MA, USA).

Column used for the analysis was Rxi®-1 MS column (60 m × 0.25 mm × 0.25 μm film thickness, Restek, Bellefonte, PA, USA). The mass-spectrometer temperatures were as follows: transfer line 280 °C and ion source 220 °C with the scanning range 32 – 450 atomic mass units (amu). The oven was programmed to heat up to 50 °C, hold there for 3 min, then perform a 4 °C/min ramp to 300 °C, hold for 10 min. The injection quantity was 0.4 μL. The identification of the compounds was based on electron spectra of the NIST 2011 library and on the Kovats retention indices.

Results:

Fig. S3. The total ion chromatogram of fennel essential oil

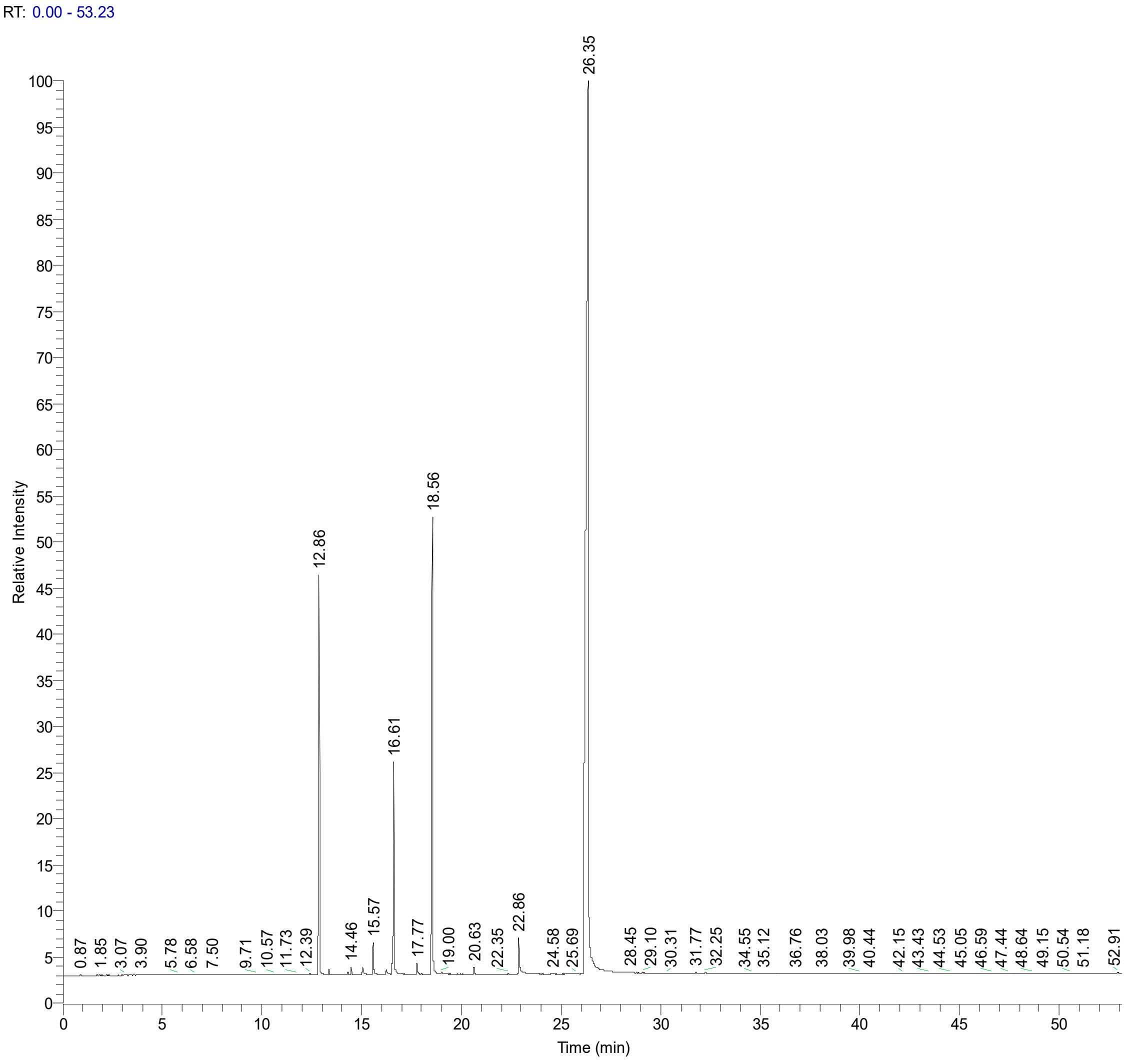

Table S7. Constituents of fennel essential oil (%)

Number	RT	RA%	Compounds
1	12.86	9.91 ± 0.11	α-Pinene
2	13.36	0.24 ± 0.01	Camphene
3	14.46	0.33 ± 0.02	β-Pinene
4	16.00	0.82 ± 0.03	α-Phellandrene
5	16.54	0.39 ± 0.02	Isocarvestrene
6	16.61	5.46 ± 0.08	Limonene
7	18.56	15.70 ± 0.14	Fenchone
8	20.63	0.18 ± 0.00	Camphor
9	22.86	1.08 ± 0.05	Estragole
10	26.35	65.40 ± 0.17	Anethole

Means are averaged values of three replicates ± standard deviation. RT – retention time; RA% - relative average content in %

Fig. S4. The total ion chromatogram of spruce essential oil

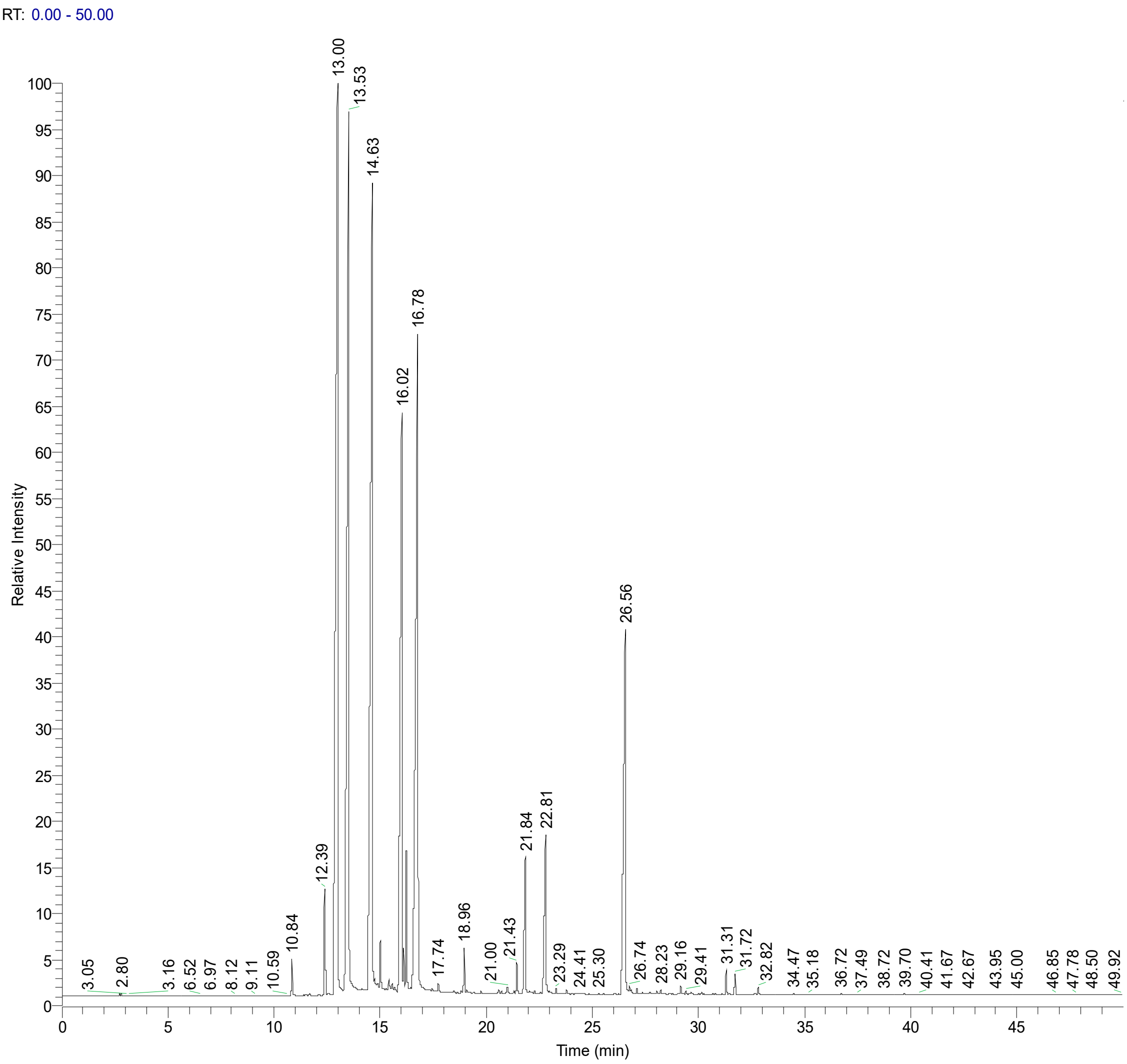

Table S8. Constituents of spruce essential oil (%)

Number	RT	RA%	Compounds
1	10.84	0.34 ± 0.02	Santene
2	12.39	1.45 ± 0.04	Cyclofenchene
3	13.00	19.21 ± 0.09	α-Pinene
4	13.53	17.81 ± 0.08	Camphene
5	14.63	16.68 ± 0.09	β-Pinene
6	15.01	0.51 ± 0.04	Myrcene
7	15.57	0.12 ± 0.00	α-Phellandrene
8	16.02	11.80 ± 0.06	δ-3-Carene
9	16.11	0.29 ± 0.02	α-Terpinene
10	16.24	1.60 ± 0.03	*o*-Cymene
11	16.78	13.93 ± 0.08	Limonene
12	17.74	0.09 ± 0.00	γ-Terpinene
13	18.96	0.46 ± 0.04	Terpinolene
14	21.43	0.41 ± 0.03	Isoborneol
15	21.84	2.29 ± 0.06	Endoborneol
16	22.81	3.12 ± 0.07	Terpineol
17	26.56	7.76 ± 0.09	Bornyl acetate
18	31.31	0.28 ± 0.02	Longifolene
19	31.72	0.22 ± 0.02	Caryophyllene
20	32.70	0.08 ± 0.00	Humulene

Means are averaged values of three replicates ± standard deviation. RT – retention time; RA% - relative average content in %
